# Synthesis and Biological Evaluation of Some Novel Thiazole-Based Heterocycles as Potential Anticancer and Antimicrobial Agents

**DOI:** 10.3390/molecules24030539

**Published:** 2019-02-01

**Authors:** Sraa Abu-Melha, Mastoura M. Edrees, Heba H. Salem, Nabila A. Kheder, Sobhi M. Gomha, Mohamad R. Abdelaziz

**Affiliations:** 1 Department of Chemistry, Faculty of Science, King Khalid University, Abha 61413, Saudi Arabia; 2 Department of Organic Chemistry, National Organization for Drug Control and Research (NODCAR), Giza 12311, Egypt; 3 Faculty of Pharmacy, King Khalid University, Abha 61441, Saudi Arabia; heba_hamedsalem@hotmail.com; 4 Department of Biochemistry, Faculty of Pharmacy, Cairo University, Cairo 11562, Egypt; 5 Department of Chemistry, Faculty of Science, Cairo University, Giza 12613, Egypt; nabila.abdelshafy@gmail.com (N.A.K.); s.m.gomha@gmail.com (S.M.G.); 6 Department of Pharmaceutical Chemistry, Faculty of Pharmacy, King Khalid University, Abha 61441, Saudi Arabia; 7 Department of Chemistry, Faculty of Science, Islamic University in Almadinah Almonawara, Almadinah Almonawara 42351, Saudi Arabia; 8 Department of Pharmaceutical Chemistry, Faculty of Pharmacy, MIU University, Cairo 19648, Egypt; Mohamedvac_75@yahoo.com

**Keywords:** thiazoles, hydrazonoyl halides, hepatoprotective activity, anticancer activity, antimicrobial activity

## Abstract

A novel series of thiazole-based heterocycles was synthesized using 1,3-dipolar cycloaddition reactions in the presence of chitosan-grafted-poly(vinylpyridine) as an eco-friendly biopolymeric basic catalyst. The molecular structure of the synthesized compounds was illustrated by spectroscopic and elemental analysis. Various in vitro biological assays were performed to explore the potential antitumor, antimicrobial and hepatoprotective activities of the newly synthesized compounds. The cytotoxic activities were assessed against human hepatocellular carcinoma (HepG-2), colorectal carcinoma (HCT-116) and breast cancer (MCF-7) cell lines and results revealed that all compounds displayed antitumor activities with the chlorine-containing derivatives, **11c** and **6g**, being the most potent. The majority of the tested thiazole derivatives exhibited satisfactory antibacterial activity towards the used gram positive and gram-negative bacterial species. Moreover, many derivatives showed weak hepatoprotective activity against CCl_4_-induced hepatotoxicity.

## 1. Introduction

Synthesis of novel bioactive compounds using green methods that minimize the use and generation of hazardous substances, is a major aim for many researchers. Thiazole derivatives have gained considerable attention because of their broad biological activities that include antidiabetic, antimicrobial, anti-inflammatory, anticancer, anti-Alzheimer, antihypertensive, antioxidant and hepatoprotective activities [1,2,3,4,5,6,7,8,9,10,11,12,13,14,15,16]. In addition, many thiazole-containing drugs such as Abafungin, Alagebrium, Acotiamide, Amiphenazole, Brecanavir, Cefepime, Carumonam, and Cefmatilen are commercially available.

Cancer is regarded as one of the dominant causes of mortality nowadays. The development of new antitumor agents represents an urgent need due to the increasing problems of various, sometimes, intolerable toxic side effects of the currently marketed drugs and the evolution of resistance to their actions [17,18]. Furthermore, liver diseases are viewed as one of the highly serious health issues globally [19]. The lack of satisfactory treatment strategies for these diseases with the occurrence of different side effects upon long term therapy, raise the demand for finding out new chemical entities that offer more efficient hepatoprotection and considerable safety. Moreover, there is a continuous compelling need for the development of new antibiotics to replace the current medications that are losing their efficacy and that could have higher efficiency or a wider spectrum.

In view of these precedents and together with our research concerns of developing new convenient approaches for the synthesis of different heterocyclic systems with auspicious pharmacological activities [20,21,22,23,24,25], we present in this report an efficient synthesis of some new series of novel thiazole derivatives using chitosan-grafted-poly(vinyl pyridine) as an eco-friendly biopolymeric basic catalyst. Additionally, we have assessed a variety of biological activities for the newly synthesized compounds that demonstrated their potential antitumor, antimicrobial and hepatoprotective effectiveness.

## 2. Results and Discussion

### 2.1. Chemistry

**Scheme 1:**
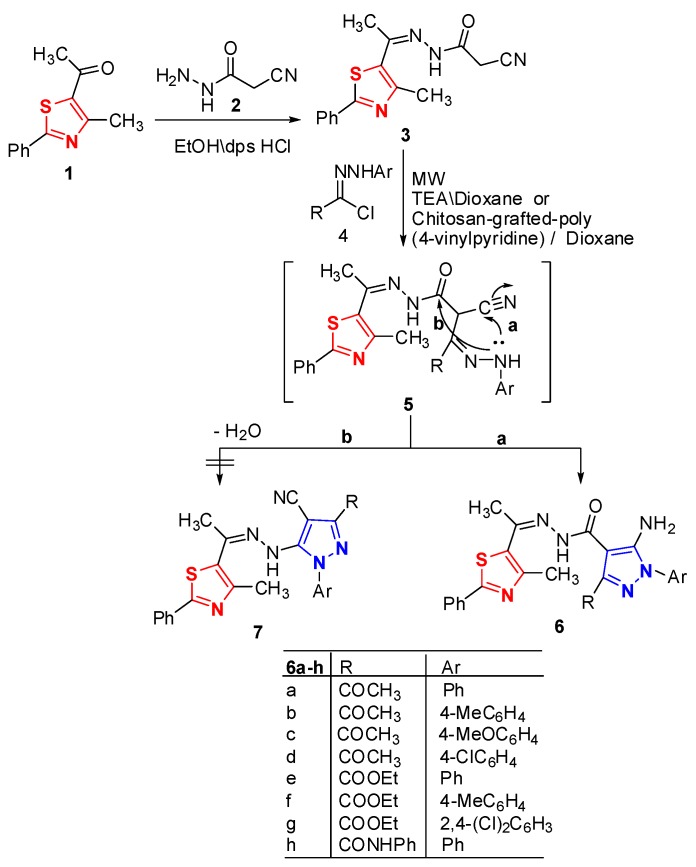
Synthesis of thiazolyl pyrazoles **6a**–**h**.

Refluxing of 5-acetyl-4-methyl-2-phenyl-thiazole (**1**) [26] and 2-cyanoacetohydrazide (**2**) [27] afforded a single product identified as 2-cyano-*N*′-(1-(4-methyl-2-phenylthiazol-5-yl)ethylidene)- acetohydrazide (**3**, Scheme 1).

Its mass spectrum was compatible with the molecular formula C_15_H_14_N_4_OS and its IR spectrum showed absorption bands at 1643, 2338, and 3430 cm^−1^ due to amido carbonyl group, cyano and NH functions, respectively. Also, its ^1^H-NMR revealed signals at δ 2.49, 2.72, 3.30 and 10.6 due to two methyl, CH_2,_ and NH protons, respectively. Moreover, its mass spectrum showed a molecular ion peak at *m*/*z* = 298.

Treatment of hydrazone derivative **3** with the appropriate hydrazonoyl halides **4a**–**h** [28,29,30,31,32] using triethylamine or chitosan as a basic catalyst and under the same experimental conditions, afforded in each case the same products which are identified as the thiazole derivatives **6a**–**h** rather than the other possible product **7** based on the spectral data (IR, MS and ^1^H-NMR) of the isolated products (Scheme 1, see Supporting Information). The distinction between the two possible products **6** and **7** was done based on the results of the spectral analysis. The IR spectra showed the absence of nitrile absorption band. Also, their ^1^H-NMR spectra revealed the presence of signals corresponds to NH_2_ protons. Moreover, their mass spectrum showed peaks corresponding to their molecular ions. The results of Table 1 indicated that high yield was obtained using chitosan as a basic catalyst.

**Scheme 2:**
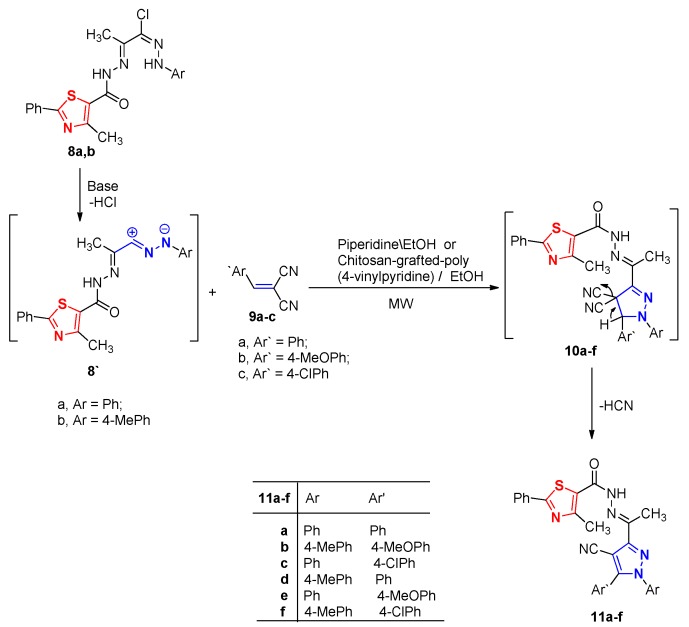
Synthesis of thiazolylpyrazoles **11a**–**f**.

Heating a mixture of hydrazonoyl halides **8a** or **8b** [23] and the appropriate arylidine malononitriles **9a**–**c** [33] in ethanol containing piperidine under irradiation by MW led to the formation of the thiazolyl pyrazoles **11a**–**f** (Scheme 2). The structure of the latter products was established based on their elemental analysis and spectral data (cf. Experimental, see Supporting Information).

When the above reaction was repeated in presence of grafted-chitosan as a catalyst and under typical reaction conditions, the same products which are identical in all aspects (m.p., mixed m.p. and IR spectra) were obtained in good yields (Table 2).

To account for the formation of the product **11**, it is suggested that the 1,3-dipolar cycloaddition of nitrile imine **8**` generated in situ from hydrazonoyl halides **8** in the presence of base) to the arylidine derivative **9** to give the intermediate **10**, followed by aromatization via losing of HCN molecule to give the final product **11** as illustrated in Scheme 2.

### 2.2. Biological Evaluation

#### 2.2.1. Cytotoxic Activity

**Figure 1:**
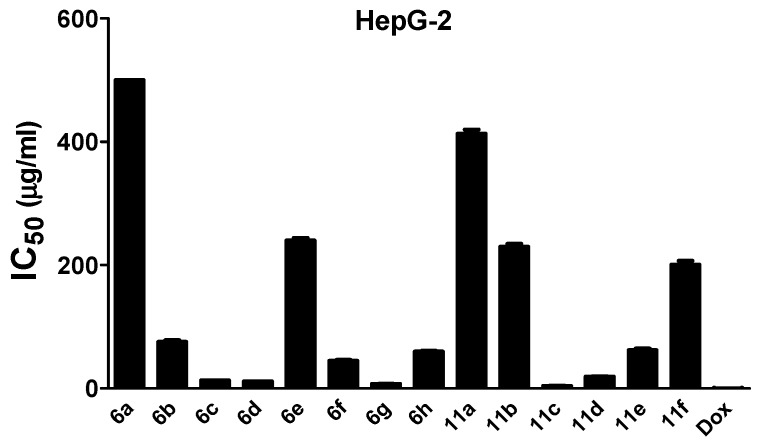
In vitro antitumor effect of synthesized thiazolyl pyrazoles **(6a**–**h**, **11a**–**f)** against HepG-2. Dox: doxorubicin, the standard drug. The analysis was performed using the MTT assay after 24 h of incubation. Values are shown as mean ± SD of three replicates. All compounds exhibited cytotoxic effects and, 11c and **6g** were the most potent. Compound **6a** has IC_50_ > 500 µg/mL.

**Figure 2:**
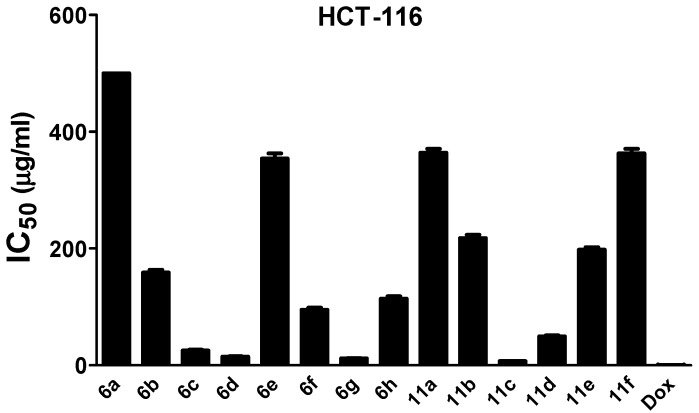
In vitro antitumor effect of synthesized thiazolyl pyrazoles (**6a**–**h**, **11a**–**f**) against HCT-116. Dox: doxorubicin, the standard drug. The analysis was performed using the MTT assay after 24 h of incubation. Values are shown as mean ± SD of three replicates. All compounds exhibited cytotoxic effects and, 11c and **6g** were the most potent. Compound **6a** has IC_50_ > 500 µg/mL.

**Figure 3:**
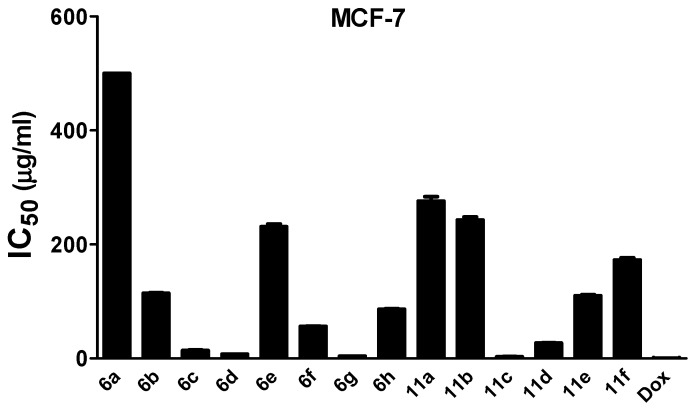
In vitro antitumor effect of synthesized thiazolyl pyrazoles (**6a**–**h**, **11a**–**f**) against MFC-7. Dox: doxorubicin, the standard drug. The analysis was performed using the MTT assay after 24 h of incubation. Values are shown as mean ± SD of three replicates. All compounds exhibited cytotoxic effects and in particular, **11c** and **6g** were the most potent. Compound **6a** has IC_50_ > 500 µg/mL.

The in vitro antitumor activity of the newly-synthesized compounds **6a**–**h** and **11a**–**f** and the reference drug, Doxorubicin was investigated against three cancer cell lines, human hepatocellular carcinoma cell line (HepG-2), colon carcinoma cells (HCT-116), and human breast carcinoma cells (MCF-7 cell line). The cytotoxic potential was determined using the MTT (methyl thiazolyl tetrazolium) assay after 24 h of incubation [34]. The concentration of the tested compounds needed to inhibit 50% of the cells (IC_50_) was calculated and presented in Table 3 and Figure 1, Figure 2 and Figure 3.

Results of the MTT assay indicated that most of investigated compounds exhibited inhibitory activity against the tested cell lines, with some derivatives showing prominent antitumor activity. Thiazole derivatives **11c** and **6g** displayed the highest cytotoxic activities against the tested cell lines with IC_50_ values of about 4 µg/mL and 7 µg/mL for HepG-2, 3 µg/mL and 4 µg/mL for MCF-7, and 7 µg/mL and 12 µg/mL, for HCT-116 cells, respectively.

According to these results, we can suggest the following structure activity relationships:

A—In the thiazolylpyrazoles **6a**–**h**:

(1) Attachment of chlorine (**6d**) or methoxy group (**6c**) at position 4 in the aryl moiety of the pyrazole ring is important for cytotoxic activity with chlorine having the higher impact in compound (**6d)**.
(2) Addition of another chlorine atom in position 2 in the aryl moiety of compound (**6g**) increases the activity which reaches the double against MCF-7 cells.

B—In the thiazolylpyrazoles **11a**–**f**:

(1) Substitution on only one of the aryl moieties of the pyrazole ring in compounds (**11c**,**d**) induces cytotoxic activity, most prominently by chlorine in compound (**11c**).
(2) Substitution on the second aryl moiety of the pyrazole ring by methyl group as in compounds (**11b**,**f**) induces great reduction (nearly abolishes) the cytotoxic activity.

#### 2.2.2. Evaluation of the Antimicrobial Activity

The in vitro antimicrobial effectiveness of the newly synthesized thiazolyl pyrazoles **6a**–**h**, **11a**–**f**, and standard drugs were investigated using the inhibition zone technique and minimum inhibitory concentration (MIC) [35,36]. The antibacterial activities were tested against the gram-positive bacteria, *Staphylococcus aureus* (CMB010010) and *Bacillus subtilis* (RCMB 010067), and the gram-negative bacteria: *Escherichia coli* (RCMB 010052) and *Proteus vulgaris* (RCMB 004 (1) ATCC 13315), while the antifungal activities were tested against *Aspergillus fumigatus* (RCMB 002008 (4)) and *Candida albicans* (RCMB 05036). Gentamycin was used as the standard antibacterial drug while ketoconazole was used as the standard antifungal drug. The results are presented in Table 4 and Table 5 and Supplementary Figures S1–S6.

The results of the antimicrobial evaluation demonstrated that all the newly synthesized thiazoles exhibited good antibacterial effect towards the gram-positive bacteria *Staphylococcus aureus* (except **11b**), and *Bacillus subtilis* (except **11f**). With regards to the gram-negative bacteria, all compounds had antibacterial activity against *Escherichia coli*, while only **6f**, **6h** and **11a**–**e** were effective against *Proteus vulgaris*. Of notice, the thiazole derivative **6f** possessed the highest antibacterial activity compared to all other tested thiazoles against *Staphylococcus aureus*, *Bacillus subtilis*, and *Escherichia coli*. Interestingly, the antimicrobial activity of this derivative approaches the potency of gentamicin, against the tested gram-negative bacteria. However, the derivative **6h** exerted the most prominent antibacterial activity against *Proteus vulgaris.* On the contrary, all synthesized compounds had no antifungal activity against *Aspergillus fumigatus* or *Candida albicans*. From these data, we can conclude that the presence of ethoxy carbonyl group and *p*-tolyl as substituents on the pyrazole ring increased the antimicrobial activity of compound **6f**.

#### 2.2.3. In Vitro Hepatoprotective Activity

The hepatoprotective potential of the newly synthesized thiazole derivatives was studied using an in vitro model of CCl_4_-induced hepatotoxicity. In vitro hepatoprotective activity was performed by assessing the viability of isolated rat hepatocytes treated with CCl_4_ in the presence and absence of the tested compounds [37]. Rat hepatocytes were isolated as previously described [38], and their viability was evaluated by the MTT reduction assay method [34,39] using silymarin as the reference standard drug. The concentration required to cure 50% of CCl_4_-exposed hepatocytes, EC_50_ was calculated and presented in Table 6. Results declared that compounds **6c**, **6d**, **6f**, **6g**, **6h**, **11c**, **11d**, and **11e** offered protection against CCl_4_-induced liver damage but lower than the standard drug. These results would suggest that these thiazole derivatives could be a candidate starting materials for the synthesis of more potent hepatoprotective drugs.

## 3. Materials and Methods

### 3.1. Chemistry

#### General Information

Melting points were measured on an Electrothermal IA 9000 series digital melting point apparatus (Bibby Sci. Lim. Stone, Staffordshire, UK). IR spectra were recorded in potassium bromide discs on PyeUnicam SP 3300 (PyeUnicam Ltd., Cambridge, UK) and FTIR 8101 PC infrared spectrophotometers (Shimadzu, Tokyo, Japan). NMR spectra were measured on a Mercury VX-300 NMR spectrometer (Varian, Inc., Karlsruhe, Germany). ^1^H-NMR spectra were recorded at 300 MHz and ^13^C-NMR spectra were recorded at 75.46 MHz in deuterated dimethyl sulfoxide (DMSO-*d*_6_). Mass spectra were run on a Shimadzu GCMS-QP1000 EX mass spectrometer (Tokyo, Japan) at 70 eV. Elemental analyses were measured using Elementarvario LIII CHNS analyzer (GmbH & Co.KG, Hanau, Germany). Biological activities of the synthesized compounds were carried out at the Regional Center for Mycology and Biotechnology at Al-Azhar University, Cairo, Egypt. Irradiation was done in a domestic microwave oven (2500 MHz, 400 W). The reactions were carried out in a closed Teflon vessel which was placed at the center of the oven for irradiation. 5-Acetyl-4-methyl-2-phenyl-thiazole (**1**) [26], 2-cyanoacetohydrazide (**2**) [27], hydrazonoyl halides **4a** [28,29], **4b**–**d** [30], **4e**–**g** [31], **4h** [32], **8a, b** [23] and arylidine malononitriles **9a**–**c** [31] were prepared as described in the literature.

Synthesis of 2-cyano-*N*′-(1-(4-methyl-2-phenylthiazol-5-yl)ethylidene)acetohydrazide (**3**). A mixture of 5-acetyl-4-methyl-2-phenyl-thiazole **1** (2.17 g, 10 mmol) and 2-cyanoacetohydrazide **2** (0.99 g, 10 mmol) in 50 mL of EtOH containing catalytic amounts of HCl was refluxed for 6 h as monitored by TLC. The precipitated solid product was filtered, washed with ethanol and recrystallized from acetic acid to give pure product of thiazole derivative **3** as white solid (81%); mp 201–203 °C; IR (KBr) *ν* 3430 (NH), 3060, 2923 (C–H), 2338 (C≡N), 1643 (C=O), 1599 (C=N) cm^−1^; ^1^H-NMR (DMSO-*d*6): δ 2.49 (s, 3H, CH_3_), 2.72 (s, 3H, CH_3_), 3.30 (s, 2H, CH_2_), 7.51–8.03 (m, 5H, Ar-H), 10.60 (s, br, 1H, NH); MS *m*/*z* (%) 298 (M^+^, 83), 217 (96), 202 (100), 174 (53), 104 (69), 64 (72). Anal. Calcd: for C_15_H_14_N_4_OS (298.36): C, 60.38; H, 4.73; N, 18.78. Found: C, 60.45; H, 4.81; N, 18.66%.

General method for synthesis of 5-amino-1-aryl-3-substituted-*N*′-(1-(4-methyl-2-phenyl thiazol-5-yl) ethylidene)-1*H*-pyrazole-4-carbohydrazides **6a**–**h**.

Method A. A mixture of hydrazone **3** (0.298 g, 1 mmol) and the appropriate hydrazonoyl halides **4** (1 mmol) in dioxane (20 mL) containing TEA (0.07 mL) was irradiated by MW at 400 Watt in a closed Teflon vessel until all the starting material was consumed (6–10 min as monitored by TLC). The hot reaction mixture was allowed to cool to room temperature and the precipitated solid was filtered off, washed with EtOH, dried and recrystallized from the suitable solvent to give the corresponding thiazole derivatives **6a**–**h**.

Method B. A mixture of hydrazone **3** (0.298 g, 1 mmol) and the appropriate hydrazonoyl halides **4** (1 mmol) in dioxane (20 mL) containing grafted-chitosan (0.1 g) was irradiated by MW at 400 Watt in a closed Teflon vessel until all the starting material was consumed (6–10 min as monitored by TLC). The hot solution was filtered to remove grafted-chitosan and excess solvent was removed under reduced pressure. The reaction mixture was triturated with methanol and the product separated was filtered, washed with methanol, dried and recrystallized from the proper solvent to give the corresponding products, **6a**–**h** which were identical in all aspects (m.p., mixed m.p. and IR spectra) with those obtained from method A**.** The physical constants of products **6a**–**h** are provided below:

*3-Acetyl-5-amino-N′-(1-(4-methyl-2-phenylthiazol-5-yl)ethylidene)-1-phenyl-1H-pyrazole-4-carbohydrazide* (**6a**). Yellow solid; mp 163–165 °C (EtOH); IR (KBr) *ν* = 3432, 3264 (NH_2_ and NH), 3056, 2998, 2924 (C–H), 1694, 1643 (2C=O), 1601 (C=N) cm^−1^; ^1^H-NMR (300 MHz, DMSO-*d*_6_) δ 2.49 (s, 3H, CH_3_), 2.58 (s, 3H, CH_3_), 2.71 (s, 3H, CH_3_), 7.50–8.01 (m, 12H, Ar-H and NH_2_), 10.63 (s, br, 1H, NH); ^13^C-NMR (DMSO-*d*_6_) δ 16.80, 18.38, 25.25 (CH_3_), 113.00, 119.78, 120.51, 125.51, 127.53, 127.56, 128.32, 128.38, 130.14, 130.67, 136.11, 136.63, 143.46, 145.17, 145.58 (Ar-C and C=N), 167.58, 184.58 (C=O) ppm; MS, *m*/*z* (%) 458 (M^+^, 37), 390 (66), 329 (78), 80 (100), 64 (70). Anal. calcd for C_24_H_22_N_6_O_2_S (458.54): C, 62.86; H, 4.84; N, 18.33. Found: C, 62.77; H, 4.81; N, 18.24%.

*3-Acetyl-5-amino-N′-(1-(4-methyl-2-phenylthiazol-5-yl)ethylidene)-1-(p-tolyl)-1H-pyrazole-4-carbo-hydrazide* (**6b**). Yellow solid; mp 181–183 °C (EtOH); IR (KBr) *ν* = 3422, 3255 (NH_2_ and NH), 3059, 2920 (C–H), 1698, 1646 (2C=O), 1601 (C=N) cm^−1^; ^1^H-NMR (300 MHz, DMSO-*d*_6_) δ 2.28 (s, 3H, CH_3_), 2.49 (s, 3H, CH_3_), 2.63 (s, 3H, CH_3_), 2.75 (s, 3H, CH_3_), 7.44–8.00 (m, 11H, Ar-H and NH_2_), 10.59 (s, br, 1H, NH); ^13^C-NMR (DMSO-*d*_6_) δ 16.82, 18.30, 20.61, 25.25 (CH_3_), 114.80, 119.35, 121.83, 125.69, 127.00, 127.83, 128.37, 129.14, 132.36, 133.07, 136.49, 137.19, 143.49, 144.92, 146.41 (Ar-C and C=N), 167.25, 184.49 (C=O) ppm; MS, *m*/*z* (%) 472 (M^+^, 40), 430 (39), 214 (100), 121 (84), 71 (62). Anal. calcd for C_25_H_24_N_6_O_2_S (472.56): C, 63.54; H, 5.12; N, 17.78. Found: C, 63.37; H, 5.04; N, 17.55%.

*3-Acetyl-5-amino-1-(4-methoxyphenyl)-N′-(1-(4-methyl-2-phenylthiazol-5-yl)ethylidene)-1H-pyrazole-4-carbohydrazide* (**6c**). Yellow solid; mp 157–159 °C (EtOH); IR (KBr) *ν* = 3427, 3264 (NH_2_ and NH), 3064, 2928 (C–H), 1667, 1643 (2C=O), 1597 (C=N) cm^−1^; ^1^H-NMR (300 MHz, DMSO-*d*_6_) δ 2.12 (s, 3H, CH_3_), 2.32 (s, 3H, CH_3_), 2.64 (s, 3H, CH_3_), 3.73 (s, 3H, OCH_3_), 6.67–7.72 (m, 11H, Ar-H and NH_2_), 10.73 (s, br, 1H, NH); ^13^C-NMR (DMSO-*d*_6_) δ 17.03, 18.35, 25.58, 53.90 (CH_3_), 113.91, 119.04, 120.82, 123.94, 126.80, 127.06, 129.32, 129.74, 130.26, 132.37, 135.27, 137.04, 142.91, 143.48, 145.18 (Ar-C and C=N), 167.62, 184.97 (C=O) ppm; MS, *m*/*z* (%) 488 (M^+^, 51), 477 (72), 369 (84), 121 (70), 80 (71), 64 (100). Anal. calcd for C_25_H_24_N_6_O_3_S (488.56): C, 61.46; H, 4.95; N, 17.20. Found: C, 61.25; H, 4.74; N, 17.05%.

*3-Acetyl-5-amino-1-(4-chlorophenyl)-N′-(1-(4-methyl-2-phenylthiazol-5-yl)ethylidene)-1H-pyrazole-4-carbo-hydrazide* (**6d**)**.** Yellow solid; mp 214–216 °C (DMF); IR (KBr) *ν* = 3424, 3252 (NH_2_ and NH), 3064, 2966 (C–H), 1668, 1623 (2C=O), 1596 (C=N) cm^−1^; ^1^H-NMR (300 MHz, DMSO-*d*_6_) δ 2.46 (s, 3H, CH_3_), 2.64 (s, 3H, CH_3_), 2.74 (s, 3H, CH_3_), 7.31–8.12 (m, 11H, Ar-H and NH_2_), 10.77 (s, br, 1H, NH); MS, *m*/*z* (%) 494 (M^+^ + 2, 24), 492 (M^+^, 61), 440 (69), 369 (70), 212 (47), 142 (100), 127 (62), 64 (55). Anal. calcd for C_24_H_21_ClN_6_O_2_S (492.98): C, 58.47; H, 4.29; N, 17.05. Found: C, 58.26; H, 4.22; N, 16.93%.

*Ethyl 5-amino-4-(2-(1-(4-methyl-2-phenylthiazol-5-yl)ethylidene)hydrazinecarbonyl)-1-phenyl-1H-pyrazole-3-carboxylate* (**6e**). Yellow solid; mp 177–179 °C (EtOH); IR (KBr) *ν* = 3433,3270 (NH_2_ and NH), 3041, 2922 (C–H), 1737, 1640 (2C=O), 1599 (C=N) cm^−1^; ^1^H-NMR (300 MHz, DMSO-*d*_6_) δ 0.97 (t, *J* = 7.0 Hz, 3H, CH_3_CH_2_), 2.37 (s, 3H, CH_3_), 2.68 (s, 3H, CH_3_), 4.02 (q, *J* = 7.0 Hz, 2H, CH_2_CH_3_), 7.21–7.62 (m, 12H, Ar-H and NH_2_), 10.69 (s, br, 1H, NH); ^13^C-NMR (DMSO-*d*_6_) δ 12.45, 15.93, 19.21 (CH_3_), 61.46 (CH_2_), 118.14, 118.99, 119.75, 120.74, 125.87, 127.36, 127.56, 128.13, 128.62, 128.97, 130.06, 130.11, 130.68, 146.20, 146.90 (Ar-C and C=N), 160.06, 166.11 (C=O) ppm; MS, *m*/*z* (%) 488 (M^+^, 75), 462 (69), 214 (100), 121 (47), 104 (47), 80 (73), 64 (99). Anal. calcd for C_25_H_24_N_6_O_3_S (488.56): C, 61.46; H, 4.95; N, 17.20. Found: C, 61.31; H, 4.73; N, 17.08%.

*Ethyl 5-amino-4-(2-(1-(4-methyl-2-phenylthiazol-5-yl)ethylidene)hydrazinecarbonyl)-1-(p-tolyl)-1H-pyrazole-3-carboxylate* (**6f**). Pale yellow solid; mp 161–163 °C (EtOH); IR (KBr) *ν* = 3428, 3266 (NH_2_ and NH), 3061, 2919 (C–H), 1731, 1644 (2C=O), 1595 (C=N) cm^−1^; ^1^H-NMR (300 MHz, DMSO-*d*_6_) δ 1.04 (t, *J* = 6.9 Hz, 3H, CH_3_CH_2_), 2.12 (s, 3H, CH_3_), 2.34 (s, 3H, CH_3_), 2.59 (s, 3H, CH_3_), 4.15 (q, *J* = 6.9 Hz, 2H, CH_2_CH_3_), 7.27–7.55 (m, 11H, Ar-H and NH_2_), 10.68 (s, br, 1H, NH); ^13^C-NMR (DMSO-*d*_6_) δ 12.18, 16.03, 19.15, 20.73 (CH_3_), 61.83 (CH_2_), 117.93, 118.37, 119.58, 120.00, 125.69, 127.18, 128.09, 128.47, 129.36, 130.05, 130.83, 132.46, 133.04, 144.94, 146.55 (Ar-C and C=N), 161.77, 166.82 (C=O) ppm; MS, *m*/*z* (%) 502 (M^+^, 33), 408 (97), 356 (73), 217 (100), 202 (44), 104 (70), 71 (86). Anal. calcd for: C_26_H_26_N_6_O_3_S (502.59): C, 62.13; H, 5.21; N, 16.72. Found: C, 62.26; H, 5.22; N, 16.60%.

*Ethyl 5-amino-1-(2,4-dichlorophenyl)-4-(2-(1-(4-methyl-2-phenylthiazol-5-yl)ethylidene) hydrazine-carbonyl)-1H-pyrazole-3-carboxylate* (**6g**). Brown solid; mp 209–211 °C (DMF); IR (KBr) *ν* = 3429, 3253 (NH_2_ and NH), 3057, 2928 (C–H), 1738, 1643 (2C=O), 1603 (C=N) cm^−1^; ^1^H-NMR (300 MHz, DMSO-*d*_6_) δ 1.27 (t, *J* = 7.9 Hz, 3H, CH_3_CH_2_), 2.49 (s, 3H, CH_3_), 2.74 (s, 3H, CH_3_), 4.32 (q, *J* = 7.9 Hz, 2H, CH_2_CH_3_), 7.36–8.16 (m, 10H, Ar-H and NH_2_), 10.57 (s, br, 1H, NH); MS, *m*/*z* (%) 557 (M^+^, 27), 498 (60), 347 (61), 202 (100), 111 (69), 80 (78), 64 (100). Anal. calcd for C_25_H_22_Cl_2_N_6_O_3_S (557.45): C, 53.86; H, 3.98; N, 15.08. Found: C, 53.75; H, 3.91; N, 14.88%.

*5-Amino-4-(2-(1-(4-methyl-2-phenylthiazol-5-yl)ethylidene)hydrazinecarbonyl)-N,1-diphenyl-1H-pyrazole-3-carboxamide* (**6h**). White solid; mp 231–233 °C (DMF); IR (KBr) *ν* = 3426, 3239 (NH_2_ and 2NH), 3057, 2928 (C–H), 1671, 1627 (2C=O), 1598 (C=N) cm^−1^; ^1^H-NMR (300 MHz, DMSO-*d*_6_) δ 2.12 (s, 3H, CH_3_), 2.65 (s, 3H, CH_3_), 7.13–7.83 (m, 17H, Ar-H and NH_2_), 10.81 (s, br, 1H, NH), 11.15 (s, br, 1H, NH); MS, *m*/*z* (%) 535 (M^+^, 73), 498 (60), 420 (51), 369 (79), 255 (100), 134 (66), 93 (100), 77 (98). Anal. calcd for C_29_H_25_N_7_O_2_S (535.62): C, 65.03; H, 4.70; N, 18.31. Found: C, 65.09; H, 4.64; N, 18.19%.

Synthesis of *N*′-(1-(4-cyano-1,4-diaryl-1*H*-pyrazol-3-yl)ethylidene)-4-methyl-2-phenylthiazole-5-carbohydrazides **11a**–**f**.

Method A: Equimolecular mixture of 2-(2-(4-methyl-2-phenylthiazole-5-carbonyl)hydrazono)-*N*′-arylpropane hydrazonoyl chloride**s 8a**,**b** (l mmol) and the appropriate arylidine malononitriles **9a**–**c** (1 mmol) in absolute EtOH (10 mL) containing catalytic amounts of piperidine (0.50 mL) was irradiated by MW at 400 Watt in a closed Teflon vessel until all the starting material was consumed (4–8 min as monitored by TLC), then cooled to room temperature. The solid product was filtered off, washed with ethanol and recrystallized from the proper solvent to give the thiazole derivatives **11a**–**f**, respectively.

Method B: A mixture of **8a**,**b** (1 mmol) and the appropriate arylidine malononitriles **9a**–**c** (1 mmol) in absolute EtOH (10 mL) containing grafted-chitosan (0.1 g) was irradiated by MW at 400 Watt in a closed Teflon vessel until all the starting material was consumed (4–8 min as monitored by TLC). The hot solution was filtered to remove grafted-chitosan and excess solvent was removed under reduced pressure. The reaction mixture was triturated with MeOH and the product separated was filtered, washed with MeOH, dried and recrystallized from the proper solvent to give the corresponding products, **11a**–**f** which were identical in all aspects (m.p., mixed m.p. and IR spectra) with those obtained from method A**.** The physical constants of the products **11a**–**f** are listed below.

*N′-(1-(4-cyano-1,5-diphenyl-1H-pyrazol-3-yl)ethylidene)-4-methyl-2-phenylthiazole-5-carbohydrazide* (**11a**). Yellow solid; mp 201–203 °C (EtOH); IR (KBr) *ν* = 3439 (NH), 3056, 2926 (C–H), 2226 (C≡N), 1643 (C=O), 1599 (C=N) cm^−1^; ^1^H-NMR (300 MHz, DMSO-*d*_6_) δ 2.31 (s, 3H, CH_3_), 2.73 (s, 3H, CH_3_), 7.22–8.01(m, 15H, Ar-H), 10.52 (s, br, 1H, NH); ^13^C-NMR (DMSO-*d*_6_) δ 11.05, 16.93 (CH_3_), 103.73, 118.14, 118.99, 119.75, 120.74, 125.87, 127.36, 127.56, 128.13, 128.62, 128.97, 130.06, 130.11, 130.68, 131.58, 145.17, 145.56, 151.93, 156.20, 156.90 (Ar-C and C=N), 170.83 (C=O) ppm; MS, *m*/*z* (%) 502 (M^+^, 15), 462 (30), 273 (41), 202 (29), 80 (100), 64 (89). Anal.Calcd for C_29_H_22_N_6_OS (502.59): C, 69.30; H, 4.41; N, 16.72. Found C, 69.17; H, 4.27; N, 16.55%.

*N′-(1-(4-Cyano-5-(4-methoxyphenyl)-1-(p-tolyl)-1H-pyrazol-3-yl)ethylidene)-4-methyl-2-phenylthiazole-5-carbohydrazide* (**11b**). Yellow solid; mp 227–229 °C (EtOH); IR (KBr) *ν* = 3313 (NH), 3041, 2917 (C–H), 2229 (C≡N), 1645 (C=O), 1588 (C=N) cm^−1^; ^1^H-NMR (300 MHz, DMSO-*d*_6_) δ 2.30 (s, 3H, CH_3_), 2.49 (s, 3H, CH_3_), 2.70 (s, 3H, CH_3_), 3.77 (s, 3H, OCH_3_), 7.16–7.73 (m, 13H, Ar-H), 10.27 (s, br, 1H, NH); ^13^C-NMR (DMSO-*d*_6_) δ 11.28, 17.16, 20.37, 54.06 (CH_3_), 102.93, 117.91, 119.38, 119.93, 121.49, 124.82, 126.66, 127.31, 128.00, 128.85, 129.48, 130.83, 131.46, 132.84, 135.08, 144.32, 145.59, 150.13, 153.91, 156.15 (Ar-C and C=N), 171.01 (C=O) ppm; MS, *m*/*z* (%) 546 (M^+^, 31), 479 (60), 399 (47), 338 (69), 149 (36), 80 (100). Anal.Calcd for C_31_H_26_N_6_O_2_S (546.64): C, 68.11; H, 4.79; N, 15.37. Found C, 68.04; H, 4.72; N, 15.25%.

*N′-(1-(5-(4-Chlorophenyl)-4-cyano-1-phenyl-1H-pyrazol-3-yl)ethylidene)-4-methyl-2-phenylthiazole-5-carbohydrazide* (**11c**).Yellow solid; mp 237–239 °C (DMF); IR (KBr) *ν* = 3426 (NH), 3057, 2930 (C–H), 2190 (C≡N), 1642 (C=O), 1606 (C=N) cm^−1^; ^1^H-NMR (300 MHz, DMSO-*d*_6_) δ 2.50 (s, 3H, CH_3_), 2.73 (s, 3H, CH_3_), 7.37–7.92 (m, 14H, Ar-H), 10.31 (s, br, 1H, NH); MS, *m*/*z* (%) 539 (M^+^ + 2, 6), 537 (M^+^, 23), 429 (55), 315 (40), 399 (47), 338 (69), 202 (100), 174 (51), 64 (73). Anal. Calcd for C_29_H_21_ClN_6_OS (537.03): C, 64.86; H, 3.94; N, 15.65. Found C, 64.66; H, 3.79; N, 15.47%.

*N′-(1-(4-Cyano-5-phenyl-1-(p-tolyl)-1H-pyrazol-3-yl)ethylidene)-4-methyl-2-phenylthiazole-5-carbohydrazide (***11d**).Yellow solid; mp 206–208 °C (EtOH); IR (KBr) *ν* = 3434 (NH), 3045, 2924 (C–H), 2229 (C≡N), 1644 (C=O), 1600 (C=N) cm^−1^; ^1^H-NMR (300 MHz, DMSO-*d*_6_) δ 2.28 (s, 3H, CH_3_), 2.50 (s, 3H, CH_3_), 2.72 (s, 3H, CH_3_), 7.29–7.63 (m, 14H, Ar-H), 10.19 (s, br, 1H, NH); ^13^C-NMR (DMSO-*d*_6_) δ 11.06, 16.94, 20.16 (CH_3_), 102.38, 117.36, 118.43, 119.56, 120.94, 122.84, 125.39, 127.06, 127.83, 128.29, 128.45, 130.17, 131.26, 132.39, 134.90, 143.79, 145.17, 149.85, 152.31, 155.96 (Ar-C and C=N), 170.92 (C=O) ppm; MS, *m*/*z* (%) 516 (M^+^, 16), 472 (31), 327 (28), 299 (27), 202 (100), 174 (44), 71 (62). Anal. Calcd for C_30_H_24_N_6_OS (516.62): C, 69.75; H, 4.68; N, 16.27. Found C, 69.69; H, 4.60; N, 16.11%.

*N′-(1-(4-Cyano-5-(4-methoxyphenyl)-1-phenyl-1H-pyrazol-3-yl)ethylidene)-4-methyl-2-phenythiazole-5-carbohydrazide* (**11e**). Yellow solid; mp 191–193 °C (EtOH); IR (KBr) *ν* = 3435 (NH), 3057, 2965 (C–H), 2196 (C≡N), 1643 (C=O), 1605 (C=N) cm^−1^; ^1^H-NMR (300 MHz, DMSO-*d*_6_) δ 2.37 (s, 3H, CH_3_), 2.68 (s, 3H, CH_3_), 3.82 (s, 3H, OCH_3_), 6.96-7.58 (m, 14H, Ar-H), 10.68 (s, br, 1H, NH); ^13^C-NMR (DMSO-*d*_6_) δ 12.13, 17.72 (CH_3_), 52.10 (OCH_3_), 105.91, 119.97, 120.76, 124.19, 124.69, 127.40, 128.80, 129.68, 130.10, 130.87, 135.22, 137.33, 142.13, 145.06, 146.48, 150.06, 150.48, 155.20, 157.33, 160.18 (Ar-C and C=N), 170.20 (C=O) ppm; MS, *m*/*z* (%) 532 (M^+^, 17), 425 (29), 311 (23), 202 (100), 174 (37), 64 (49). Anal. Calcd for C_30_H_24_N_6_O_2_S (532.62): C, 67.65; H, 4.54; N, 15.78. Found C, 67.49; H, 4.46; N, 15.69%.

*N′-(1-(5-(4-Chlorophenyl)-4-cyano-1-(p-tolyl)-1H-pyrazol-3-yl)ethylidene)-4-methyl-2-phenylthiazole-5-carbohydrazide* (**11f**). Yellow solid; mp 237–239 °C (DMF); IR (KBr) *ν* = 3431 (NH), 3040, 2926 (C–H), 2197 (C≡N), 1641 (C=O), 1602 (C=N) cm^−1^; ^1^H-NMR (300 MHz, DMSO-*d*_6_) δ 2.32 (s, 3H, CH_3_), 2.50 (s, 3H, CH_3_), 2.76 (s, 3H, CH_3_), 7.31–7.83 (m, 13H, Ar-H), 10.38 (s, br, 1H, NH); MS, *m*/*z* (%) 553 (M^+^ + 2, 3), 551 (M^+^, 12), 380 (53), 202 (100), 109 (48), 64 (70). Anal.Calcd for C_30_H_23_ClN_6_OS (551.06): C, 65.39; H, 4.21; N, 15.25. Found C, 65.22; H, 4.15; N, 15.10%.

### 3.2. Biological Assays

#### 3.2.1. In Vitro Cytotoxic Activity

The cytotoxic potential of the newly synthesized compounds was examined against three cancer cell lines HepG2, HCT-116, and MCF-7 using the MTT assay after 24 h of incubation [34]. For more details, see the Supporting Information file.

#### 3.2.2. Antimicrobial Evaluation

Antifungal and antibacterial activities of the synthesized thiazoles were assessed towards different microbes using the agar diffusion method and were compared to standard reference drugs [35,36]. Refer to the Supporting Information file for more details.

#### 3.2.3. Hepatoprotective Activity

In vitro hepatoprotective activity was done by assessing the viability of isolated rat hepatocytes exposed to 1% CCl_4_ along with or without the tested compounds [37]. Rat hepatocytes were isolated as described [38] and cell viability was evaluated by the MTT reduction assay [34,39] using silymarin as the reference standard drug. For further details, refer to the Supporting Information file.

## 4. Conclusions

We have efficiently synthesized a new series of thiazolylpyrazoles using hydrazonoyl halides and 2-cyano-*N*′-(1-(4-methyl-2-phenylthiazol-5-yl)ethylidene)acetohydrazide in the presence of chitosan-grafted-poly(vinylpyridine) as an eco-friendly biopolymeric basic catalyst. The structures of these novel compounds were determined using spectroscopic analyses (IR, NMR, and MS). All compounds were evaluated for their cytotoxic effectiveness against HepG-2, MCF-7, and HCT-116 cell lines. Our results indicated that most compounds exhibited a good anticancer activity and importantly, the thiazole derivatives **11c** and **6g** exhibited the greatest cytotoxic potential against the examined cell lines. In addition, antibacterial evaluation experiments illustrated that the thiazole derivative **6f** has the most potent activity towards *Staphylococcus aureus*, *Bacillus subtilis*, and *Escherichia coli.* The antibacterial potency of **6f** against the tested gram--positive bacteria even approaches that of gentamicin. However, the derivative **6h** exerted the highest antibacterial activity against *Proteus vulgaris.* Some of the tested compounds showed a weak hepatoprotective effect against CCl_4_-induced liver damage suggesting their usage as a candidate starting materials for the synthesis of more potent hepatoprotective drugs. Taken together, the current work presents an eco-friendly approach for the synthesis of novel thiazole derivatives that have potential values in protection against cancer cells and bacterial infections and could be used to develop effective agents to guard against liver toxicity.

## Figures and Tables

**Table 1:** Effect of nature of basic catalyst on the product yields **6a**–**h**.

No.	Time (min)	Yield %	
		TEA	g-Chitosan
**6a**	4	67	80
**6b**	6	69	82
**6c**	9	68	84
**6d**	5	73	85
**6e**	10	73	84
**6f**	8	68	83
**6g**	7	76	88
**6h**	7	73	81

**Table 2:** Effect of nature of basic catalyst on the product yields **11a**–**f**.

No.	Time (min)	Yield %	
		Piperidine	g-Chitosan
**11a**	5	69	83
**11b**	7	71	81
**11c**	5	74	86
**11d**	8	72	81
**11e**	3	71	84
**11f**	7	73	85

**Table 3:** Cytotoxic activity of the synthesized thiazolyl pyrazoles against HepG-2, HCT-116, and MCF-7 cell lines, expressed as IC_50_ values and compared to doxorubicin, the standard drug.

Tested Compounds	IC_50_ (μg/mL)		
	HepG-2	HCT-116	MCF-7
**6a**	>500	>500	>500
**6b**	75.5 ± 2.7	159 ± 4.7	114 ± 1.2
**6c**	13.1 ± 0.4	25.4 ± 1.3	13.9 ± 0.9
**6d**	11.4 ± 0.2	14.8 ± 0.6	7.36 ± 0.4
**6e**	240 ± 4.3	354 ± 8.9	231 ± 4.5
**6f**	44.8 ± 1.3	95 ± 3.8	56.1 ± 0.7
**6g**	7.4 ± 0.2	11.8 ± 0.5	3.77 ± 0.2
**6h**	60 ± 1.1	114 ± 4.1	86.2 ± 1.1
**11a**	413 ± 6.9	364 ± 6.9	276 ± 7.8
**11b**	230 ± 4.6	218 ± 5.3	243 ± 4.9
**11c**	4.24 ± 0.3	7.35 ± 0.4	2.99 ± 0.2
**11d**	19.3 ± 0.8	49.6 ± 1.7	26.8 ± 0.8
**11e**	62.1 ± 2.6	198 ± 4.2	110 ± 1.9
**11f**	201 ± 5.9	363 ± 7.8	173 ± 3.5
**Doxorubicin**	0.36 ± 0.04	0.49 ± 0.07	0.35 ± 0.03

The analysis was performed using the MTT assay after 24 h of incubation. Values are shown as mean ± SD of three replicates.

**Table 4:** Antimicrobial activities of the new thiazole derivatives **6a**–**h** and **11a**–**f** expressed as inhibition zones diameter in millimeters (mm).

Sample	Microorganisms					
	Fungi		Gram Positive Bacteria		Gram Negative Bacteria	
	*AF*	*CA*	*SA*	*BS*	*EC*	*PV*
**6a**	NA	NA	12 ± 0.6	11 ± 0.5	10 ± 0.3	NA
**6b**	NA	NA	13 ± 0.8	16 ± 0.7	12 ± 0.7	NA
**6c**	NA	NA	14 ± 0.6	15 ± 0.4	14 ± 0.4	NA
**6d**	NA	NA	12 ± 0.7	16 ± 0.9	13 ± 0.6	NA
**6e**	NA	NA	11 ± 0.4	17 ± 0.8	12 ± 0.8	NA
**6f**	NA	NA	20 ± 0.9	22 ± 1.3	17 ± 0.5	12 ± 0.9
**6g**	NA	NA	14 ± 0.6	16 ± 0.4	13 ± 0.7	NA
**6h**	NA	NA	12 ± 0.8	11 ± 0.6	16 ± 0.5	15 ± 0.7
**11a**	NA	NA	10 ± 0.7	12 ± 0.8	11 ± 0.4	10 ± 0.3
**11b**	NA	NA	NA	13 ± 0.5	9 ± 0.2	11 ± 0.4
**11c**	NA	NA	16 ± 0.4	12 ± 0.7	15 ± 0.9	13 ± 0.5
**11d**	NA	NA	14 ± 0.7	12 ± 0.4	13 ± 0.6	14 ± 0.7
**11e**	NA	NA	15 ± 0.9	11 ± 0.6	12 ± 0.7	10 ± 0.2
**11f**	NA	NA	9 ± 0.4	NA	10 ± 0.3	NA
**Ketoconazole**	17 ± 0.4	20 ± 0.8	-	-	-	-
**Gentamycin**	-	-	24 ± 1.2	26 ± 0.7	30 ± 0.9	25 ± 0.8

NA: No activity, results are shown as mean of inhibition zone diameter (mm) for different compounds done in triplicate ± SD; AF (*Aspergillus fumigatus* (RCMB 002008 (4)), *CA* (*Candida albicans* (RCMB 05036), *SA* (*Staphylococcus aureus* CMB010010)), *BS* (*Bacillus subtilis* (RCMB 010067)), *EC* (*Escherichia coli* (RCMB 010052)), *PV* (*Proteus vulgaris* RCMB 004 (1) ATCC 13315).

**Table 5:** Antimicrobial activities of the newly synthesized thiazoles **6a**–**h** and **11a**–**f** was shown as minimum inhibitory concentration (MIC) in µg/mL of the tested microorganisms.

Sample	Microorganisms					
	Fungi		Gram Positive Bacteria		Gram Negative Bacteria	
	*AF*	*CA*	*SA*	*BS*	*EC*	*PV*
**6a**	NA	NA	625	5000	5000	NA
**6b**	NA	NA	2500	312.5	625	NA
**6c**	NA	NA	312.5	1250	625	NA
**6d**	NA	NA	156.25	625	312.5	NA
**6e**	NA	NA	5000	625	1250	NA
**6f**	NA	NA	78.13	396	156.25	315
**6g**	NA	NA	312.5	78.13	625	NA
**6h**	NA	NA	1250	5000	156.25	312.5
**11a**	NA	NA	5000	2500	2500	5000
**11b**	NA	NA	NA	1250	10,000	5000
**11c**	NA	NA	312.5	1250	625	1250
**11d**	NA	NA	625	1250	1250	312.5
**11e**	NA	NA	625	2500	2500	5000
**11f**	NA	NA	10,000	NA	5000	NA

NA: No activity. Experiment was done using the diffusion agar method.

**Table 6:** In vitro hepatoprotective activities of the investigated compounds and reference standard drug, presented as EC_50_ values.

Tested Compounds	Hepatoprotective Activity (EC_50_ µg/mL)
**6a**	NA
**6b**	NA
**6c**	368 ± 14.6
**6d**	972 ± 96.2
**6e**	NA
**6f**	1350 ± 87
**6g**	456 ± 32
**6h**	1324 ± 64.6
**11a**	NA
**11b**	NA
**11c**	724 ± 31.7
**11d**	936 ± 64
**11e**	1980 ± 213
**11f**	NA
**Silymarin**	34.9 ± 0.6

NA: No Hepatoprotective activity when tested at concentrations ranged from 1 to 6000 µg/mL. Values are shown as mean ± SD of four replicates.

## Supplementary Materials

The following are available online. Online supplementary information includes detailed methods of the cytotoxic, antimicrobial and hepatoprotective evaluations, Figures (S1–S6) of mean zone of inhibition of the newly synthesized compounds. Also ^1^H- and ^13^C-NMR spectra of some of the synthesized thiazolylpyrazoles derivatives are included.
