# Supplementary material for: Synthesis and Biological Evaluation of Some Novel Thiazole-Based Heterocycles as Potential Anticancer and Antimicrobial Agents
Source: Molecules. 2019 Feb 1;24(3):539. doi: 10.3390/molecules24030539 (PMC6384564; doi:10.3390/molecules24030539)
Supplement: Supplementary file 1 [file molecules-24-00539-s001.pdf]

Synthesis and Biological Evaluation of Some Novel Thiazole-Based Heterocycles as Potential Anticancer and Antimicrobial Agents

Sraa Abu-Melha [1,*], Mastoura M. Edrees [1,2,*], Heba H. Salem [3,4], Nabila A. Kheder [5,6], Sobhi M. Gomha [5,7] and Mohamad R. Abdelaziz [8]

Supplementary Methods

Method of evaluation of the cytotoxic effects of the synthesized compounds

Mammalian cell lines: MCF-7 cells (human breast cancer cell line), HepG-2 cells (human Hepatocellular carcinoma) and HCT-116 (colon carcinoma) were obtained from VACSERA Tissue Culture Unit.

Chemicals Used: Dimethyl sulfoxide (DMSO), crystal violet and trypan blue dye were purchased from Sigma (St. Louis, Mo., USA).

Fetal Bovine serum, DMEM, RPMI-1640, HEPES buffer solution, L-glutamine, gentamycin and 0.25% Trypsin-EDTA were purchased from Lonza.

Crystal violet stain (1%): It composed of 0.5% (w/v) crystal violet and 50% methanol then made up to volume with ddH2O and filtered through a Whatmann No.1 filter paper.

Cell line Propagation:

The cells were propagated in Dulbecco's modified Eagle's medium (DMEM) supplemented with 10% heat-inactivated fetal bovine serum, 1% L-glutamine, HEPES buffer and 50 µg/ml gentamycin. All cells were maintained at 37 ℃ in a humidified atmosphere with 5% CO_2 and were subcultured two times a week.

Cytotoxicity evaluation using viability assay: For cytotoxicity assay, the cells were seeded in 96-well plate at a cell concentration of 1×104 cells per well in 100 µL of growth medium. Fresh medium

containing different concentrations of the test sample was added after 24 h of seeding. Serial two-fold dilutions of the tested chemical compound were added to confluent cell monolayers dispensed into 96-well, flat-bottomed microtiter plates (Falcon, NJ, USA) using a multichannel pipette. The microtiter plates were incubated at 37 ℃ in a humidified incubator with 5% CO_2 for a period of 24 h. Three wells were used for each concentration of the test sample. Control cells were incubated without test sample and with or without DMSO. The little percentage of DMSO present in the wells (maximal 0.1%) was found not to affect the experiment. After incubation of the cells at 37 ℃, for 24 h, the viable cells yield was determined by a colorimetric method [34].

In brief, after the end of the incubation period, media were aspirated and the crystal violet solution (1%) was added to each well for at least 30 minutes. The stain was removed and the plates were rinsed using tap water until all excess stain is removed. Glacial acetic acid (30%) was then added to all wells and mixed thoroughly, and then the absorbance of the plates were measured after gently shaken on Microplate reader (TECAN, Inc.), using a test wavelength of 490 nm. All results were corrected for background absorbance detected in wells without added stain. Treated samples were compared with the cell control in the absence of the tested compounds. All experiments were carried out in triplicate. The cell cytotoxic effect of each tested compound was calculated. The optical density was measured with the microplate reader (SunRise, TECAN, Inc, USA) to determine the number of viable cells and the percentage of viability was calculated as [(ODt/ODc)] x100% where ODt is the mean optical density of wells treated with the tested sample and ODc is the mean optical density of untreated cells. The relation between surviving cells and drug concentration is plotted to get the survival curve of each tumor cell line after treatment with the specified compound. The 50% inhibitory concentration (IC_{50}), the concentration required to cause toxic effects in 50% of intact cells, was estimated from graphic plots of the dose response curve for each conc. using Graphpad Prism software (San Diego, CA. USA).

Method of antimicrobial evaluation of the synthesized thiazole derivatives.

All microbial strains were provided from culture collection of the Regional Center for Mycology and Biotechnology (RCMB), Al-Azhar University, Cairo, Egypt. The antimicrobial activity was investigated on a newly synthesized compound in order to increase the selectivity of these derivatives towards test microorganisms using the agar diffusion method using Mueller-Hinton agar medium for bacteria and Sabouraud’s agar medium for fungi. Briefly, 100 μL of the test bacteria/fungi were grown in 10 mL of fresh media until they reached a count of approximately108 cells/ml for bacteria or 105 cells/mL for

fungi. All the newly synthesized compounds were weighed and dissolved in dimethyl sulfoxide to prepare extract stock solution. One hundred μL of each sample at 10 mg/mL was added to each well (10 mm diameter holes cut in the agar gel). The plates were incubated for 24-48 h at 37 ℃ (for bacteria and yeast) and for 48 h at 28 ℃ (for filamentous fungi). After incubation, the microorganism's growth was observed. The resulting inhibition zone diameters were measured in millimeters and used as criterion for the antimicrobial activity. The size of this clear zone is proportional to the inhibitory action of the compound under investigation. DMSO was used for dissolving the tested compounds thus used as solvent control and showed no inhibition zones, confirming that it has no influence on growth of the tested microorganisms. Positive controls were also performed using gentamycin as standard antibacterial drugs and ketoconazole as standard antifungal drug [35,36].

Hepatoprotective study in isolated rat hepatocytes using MTT assay [37]

Rat Hepatocyte Isolation:

Hepatocyte isolation was performed according to the collagenase perfusion procedure which was described by Reese and Byard [38]. Hepatocytes (1×10^6 cells/mL) were placed into Krebs-Henseleit buffer (pH: 7.4) containing 12.5 mM HEPES (Sigma-Aldrich, UK) and kept at 37 ℃ with 95% O_2 and 5% CO_2.

Experimental Design:

Rat hepatocytes were exposed to toxicant containing 1% CCL_4 along with or without 100 μl of the tested samples of different concentrations (ranging from 1 to 6000 μg/mL) or the medium alone is considered as control and was incubated at 37 ℃ in 5% CO_2 atmosphere for 24h. At the end of the period cytotoxicity was assessed by estimating the viability of hepatocytes by the MTT reduction assay. The experimental groups were as follows:

Group 1: Control, untreated hepatocytes

Group 2: Hepatocytes with 1% CCL_4

Group 2: Hepatocytes with 1% CCL_4 and tested compounds

Group 2: Hepatocytes with 1% CCL_4 and silymarin standard drug

Each treatment was repeated four times

MTT assay

The metabolic activity of living cells was assessed by the activity of dehydrogenases [34,38]. Following treatment with the abovementioned methods, after 24h the medium was removed and 50 µL of 3-(4,5-dimethylthiazol-2-yl)-2,5-diphenyltetrazolium bromide (MTT) (5 mg/ml; Sigma, St. Louis, CA, USA) was added to each well containing 100 µl from hepatocyte suspension (1×10^6 cells/mL). Four replicates were used for each treatments and corresponding controls. The plates were gently shaken then incubated in the dark at 37˚C for an additional 4 h in 5% CO_2 atmosphere. The reaction was stopped by the addition of 150 µl DMSO (Sigma) and the absorbance of samples at 570 nm was measured with a microplate reader (SunRise, Tecan, USA). The tetrazolium salt (3-(4,5–dimethylthiazole-2-yl)-2,5 diphenyl tetrazolium bromide, MTT) is introduced into cells and reduced in a mitochondria dependent reaction to yield a blue colored formazan product. The product accumulates within the cell due to the fact that it cannot pass through the plasma membrane. On solubilization of the cells, the product is liberated and can be readily detected and quantified by a simple colorimetric method. The ability of cells to reduce MTT provides an indication of mitochondrial integrity and activity, which in turn may be interpreted as a measure of viability and/or number of cells. The assay has therefore been adopted for use with cultures of exponentially growing cells. Determination of the cell ability to reduce MTT to the formazan derivative after exposure to test compounds shows hepatoprotective effect. The optical density of the formazan formed in the control cells was taken as 100%. The viability of hepatocytes in other groups was presented as a percentage of the control cells.

Data Analysis:

The results were expressed as:

The percentage of viability was calculated as [(ODt/ODc)] x100% where ODt is the mean optical density of wells treated with the tested sample and ODc is the mean optical density of untreated cells.

Hepatoprotective Percent = % Viability of treatment group – % Viability of negative control.

The 50% Effective concentration (EC_{50}), the concentration required to cure 50% of intact cells, was estimated from graphic plots of the dose response curve for each conc. using Graphpad Prism software (San Diego, CA. USA).

<u>Supplementary Figures:</u>

Figures of mean zone of inhibition of the newly synthesized pyrazolines

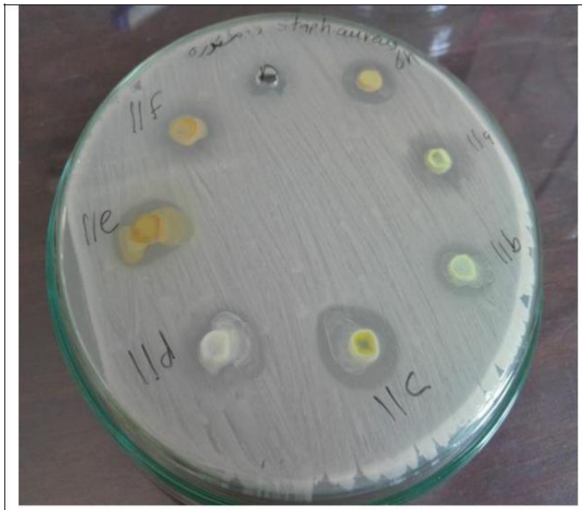

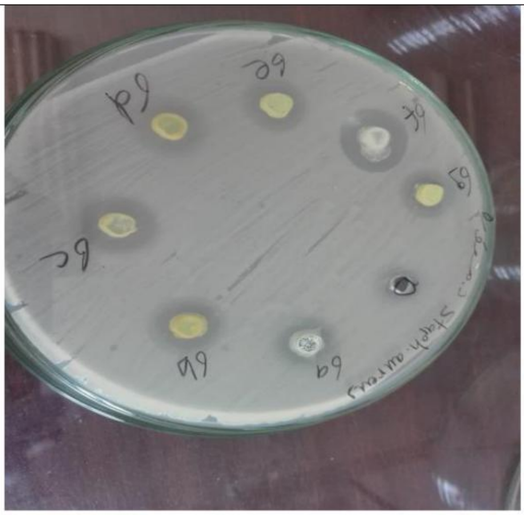

Fig S1. Mean zone of inhibition of the newly synthesized thiazoles tested against *Staphylococcus aureus* (RCMB010010).

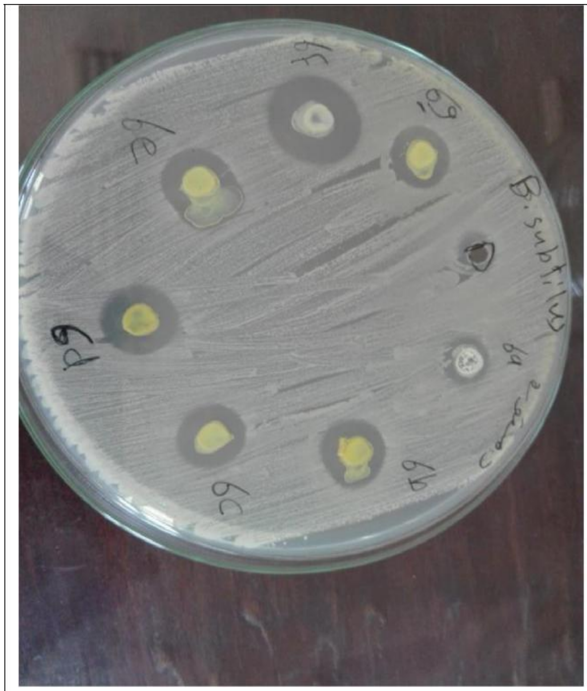

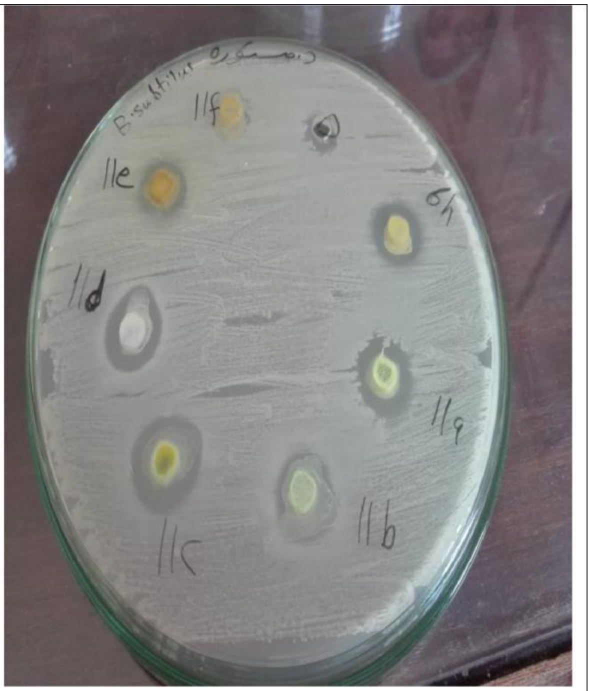

Fig S2. Mean zone of inhibition of the newly synthesized thiazoles tested against *Bacillus subtilis* (RCMB 010067)

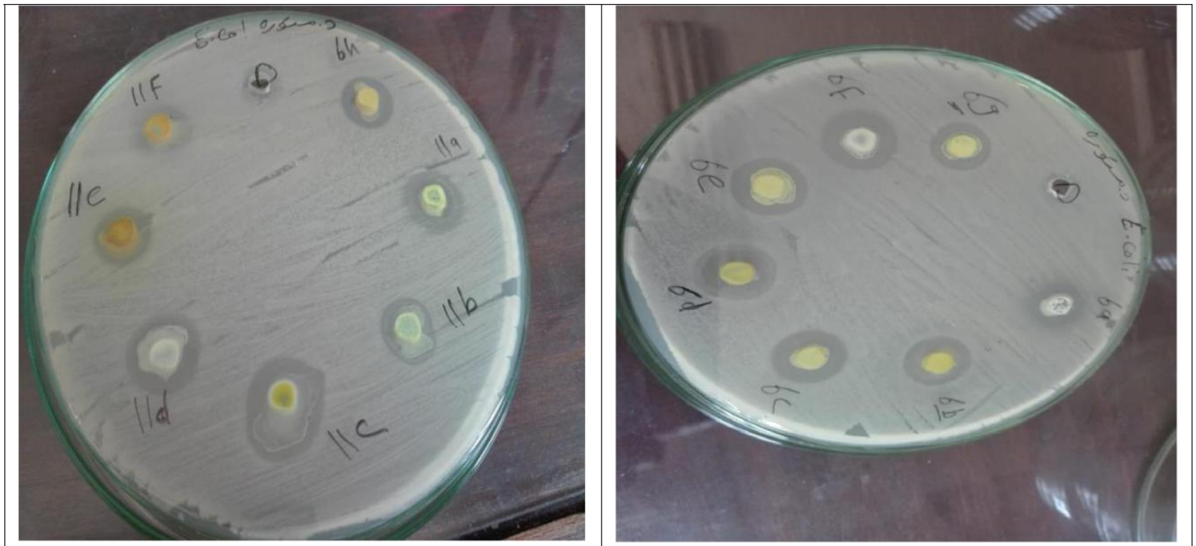

Fig S3. Mean zone of inhibition of the newly synthesized thiazoles tested against *Escherichia coli* (RCMB 010052).

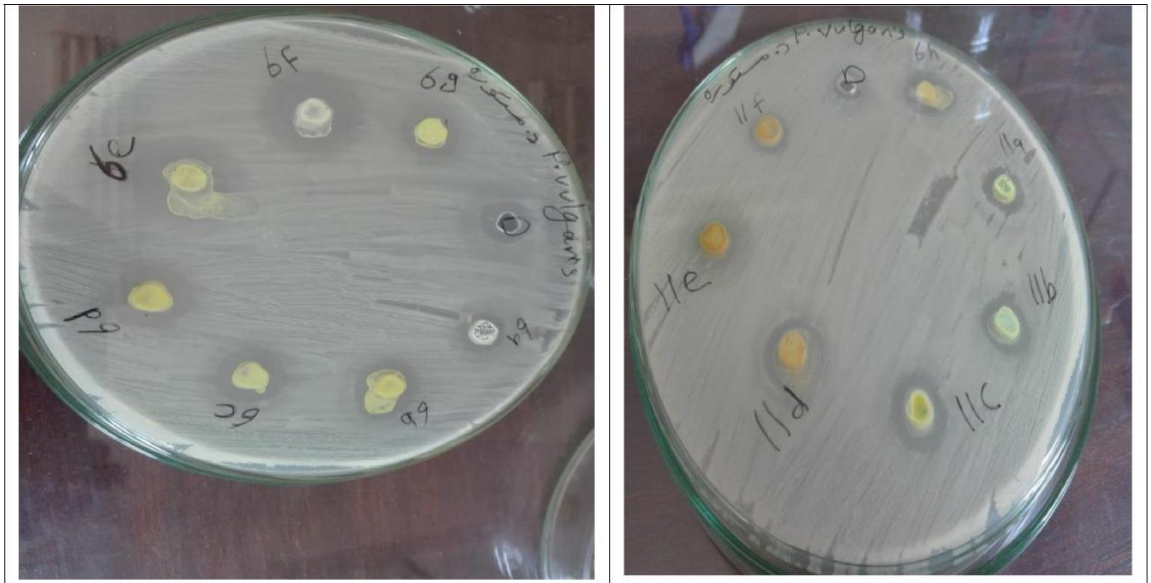

Fig S4. Mean zone of inhibition of the newly synthesized thiazoles tested against *Proteus vulgaris* RCMB 004 (1) ATCC 13315

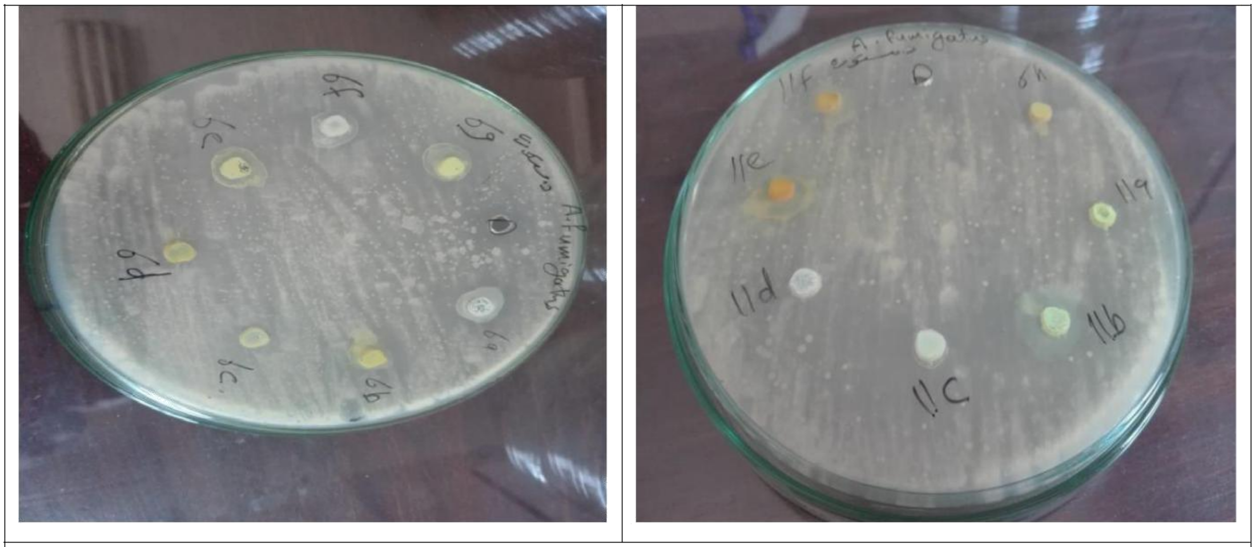

Fig. S5: Mean zone of inhibition of the newly synthesized thiazoles tested against *Aspergillus fumigatus* (RCMB 002008 (4).

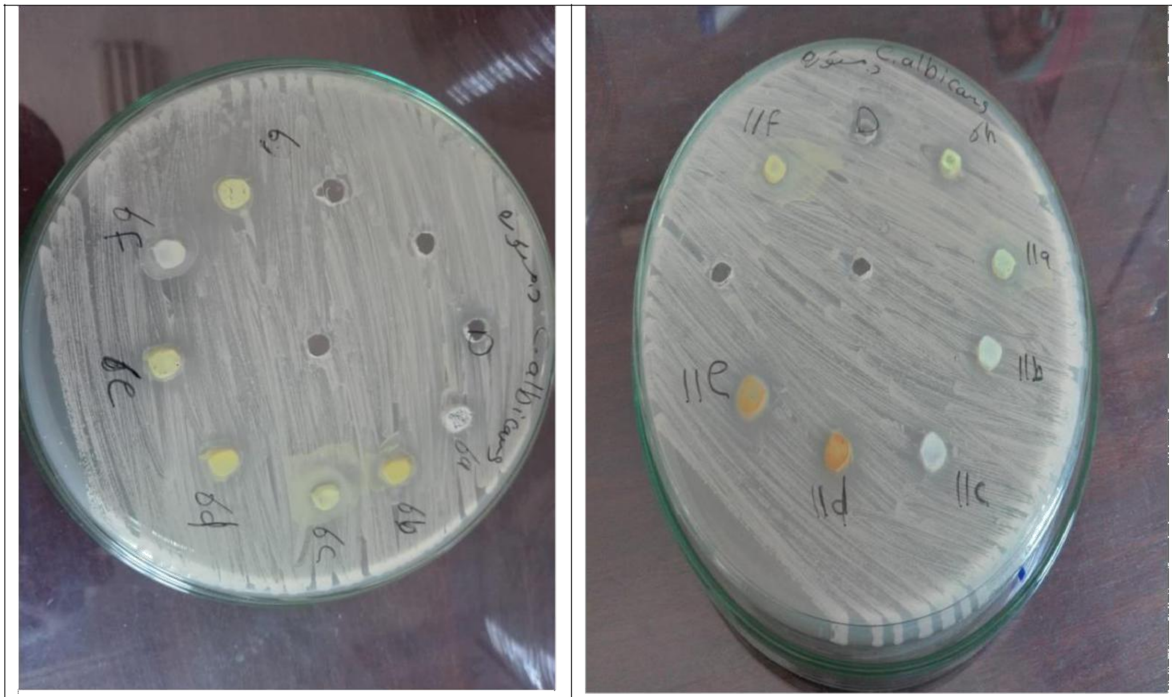

Fig. S6: Mean zone of inhibition of the newly synthesized thiazoles tested against *Candida albicans* (RCMB 05036).

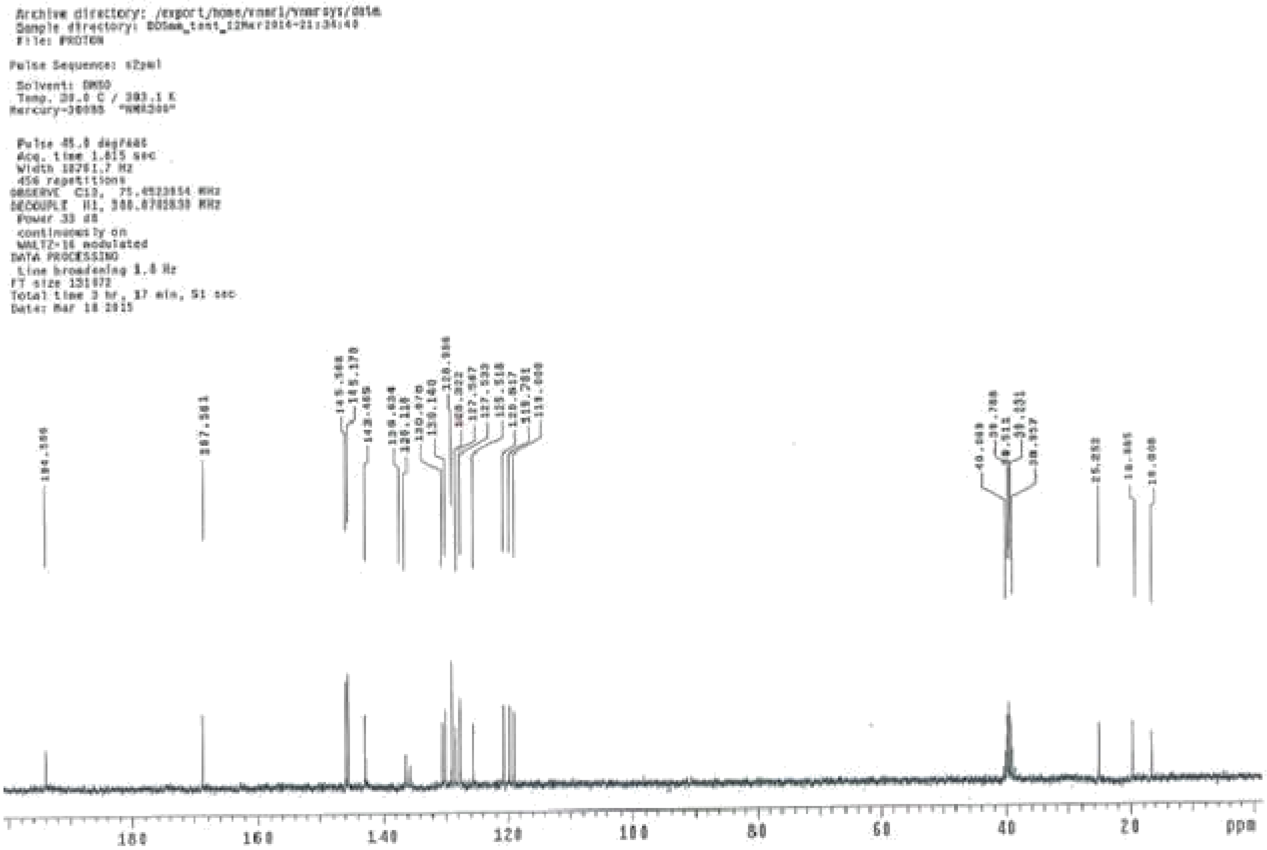

^{13}C NMR spectrum of compound 6a

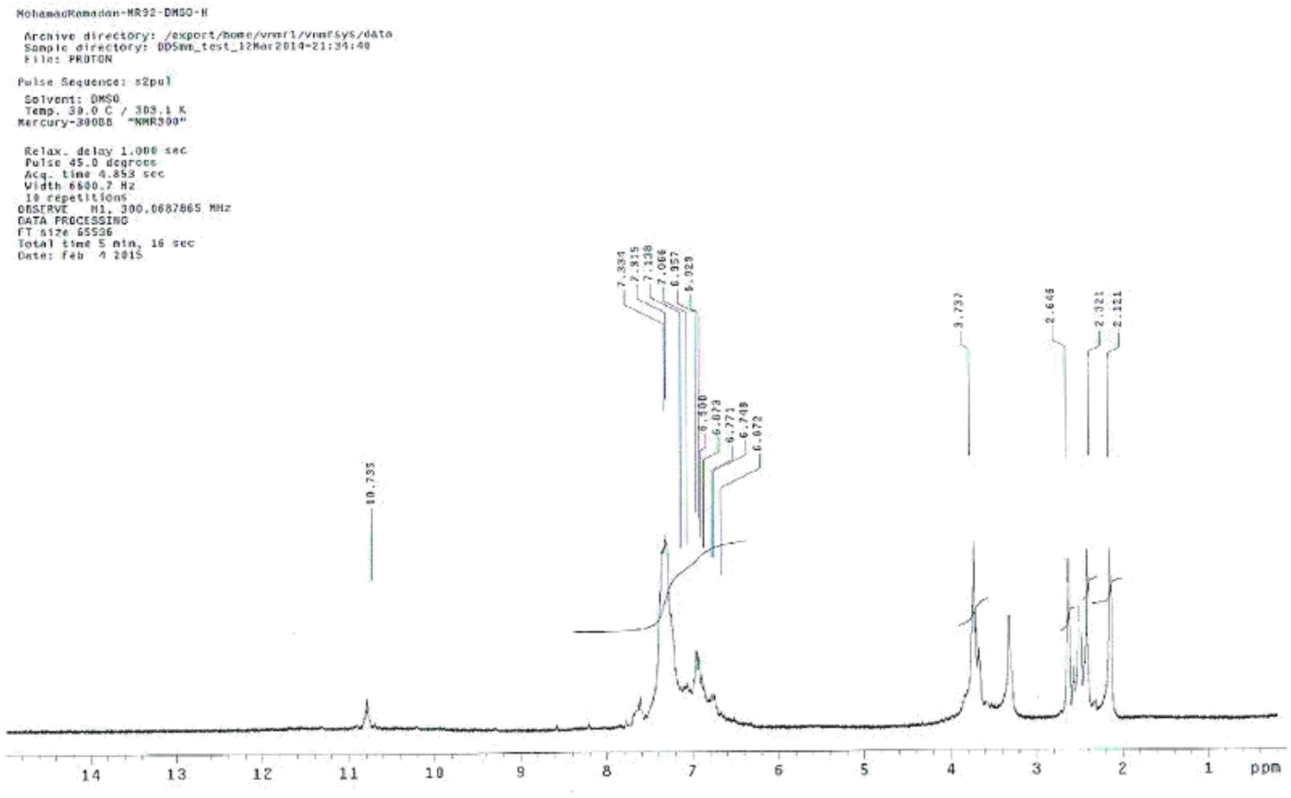

^{1}H NMR spectrum of compound 6c

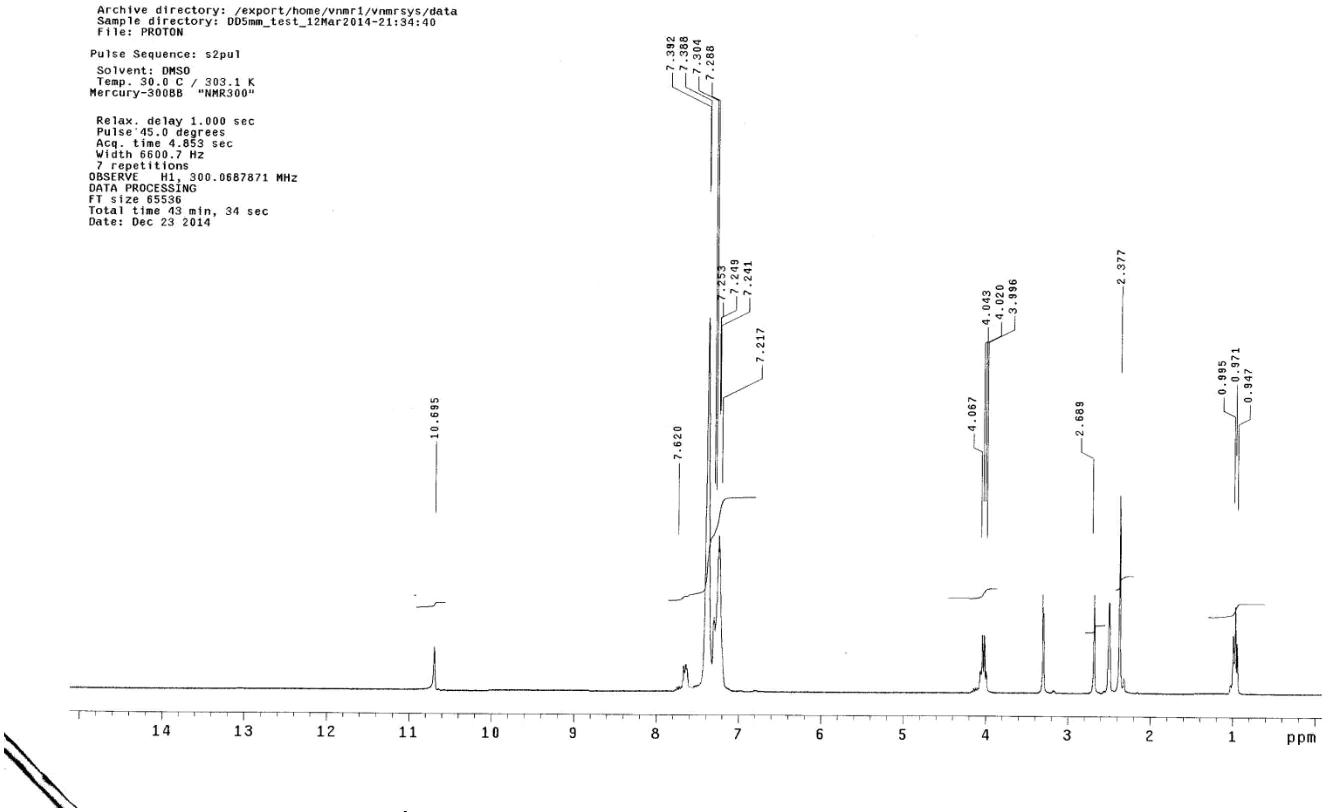

^{1}H NMR spectrum of compound 6e

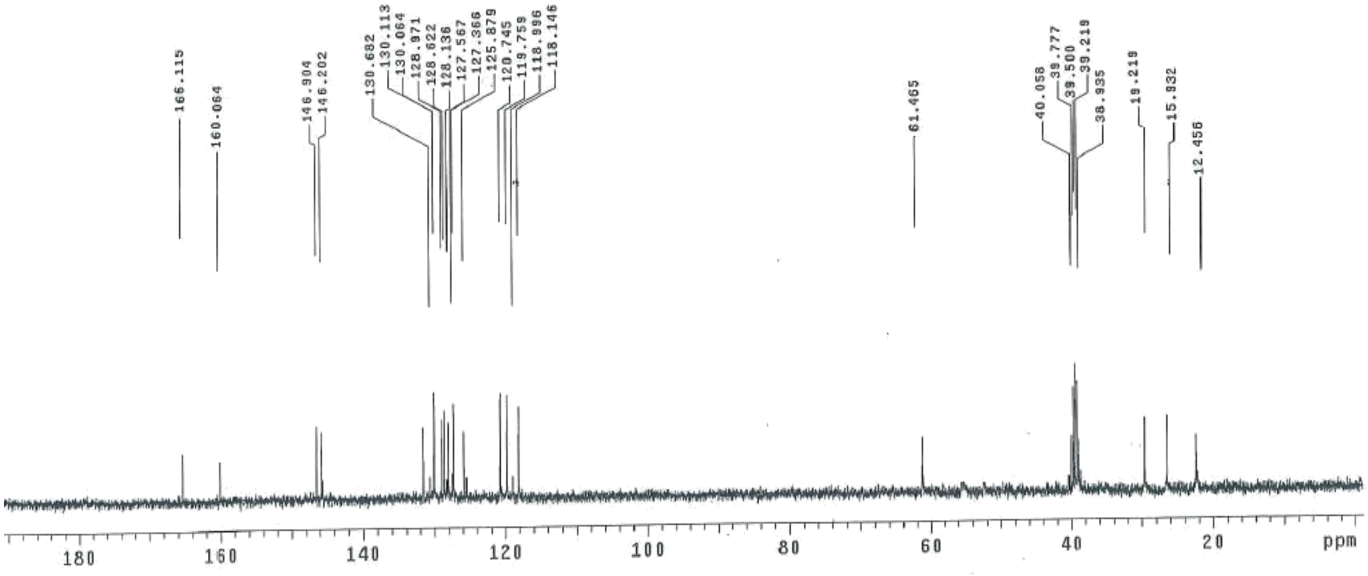

^{13}C NMR spectrum of compound 6e

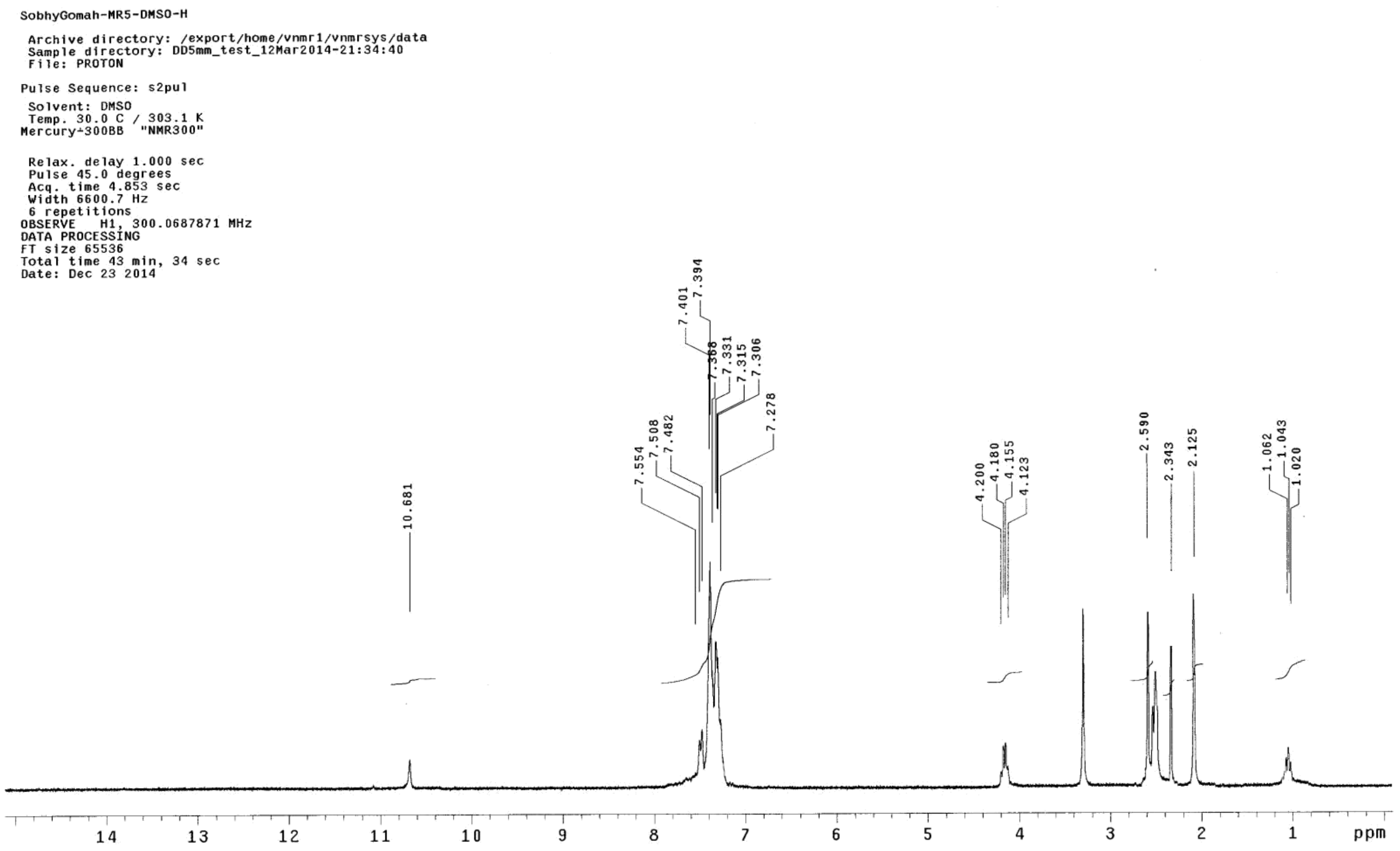

^{1}H NMR spectrum of compound 6f

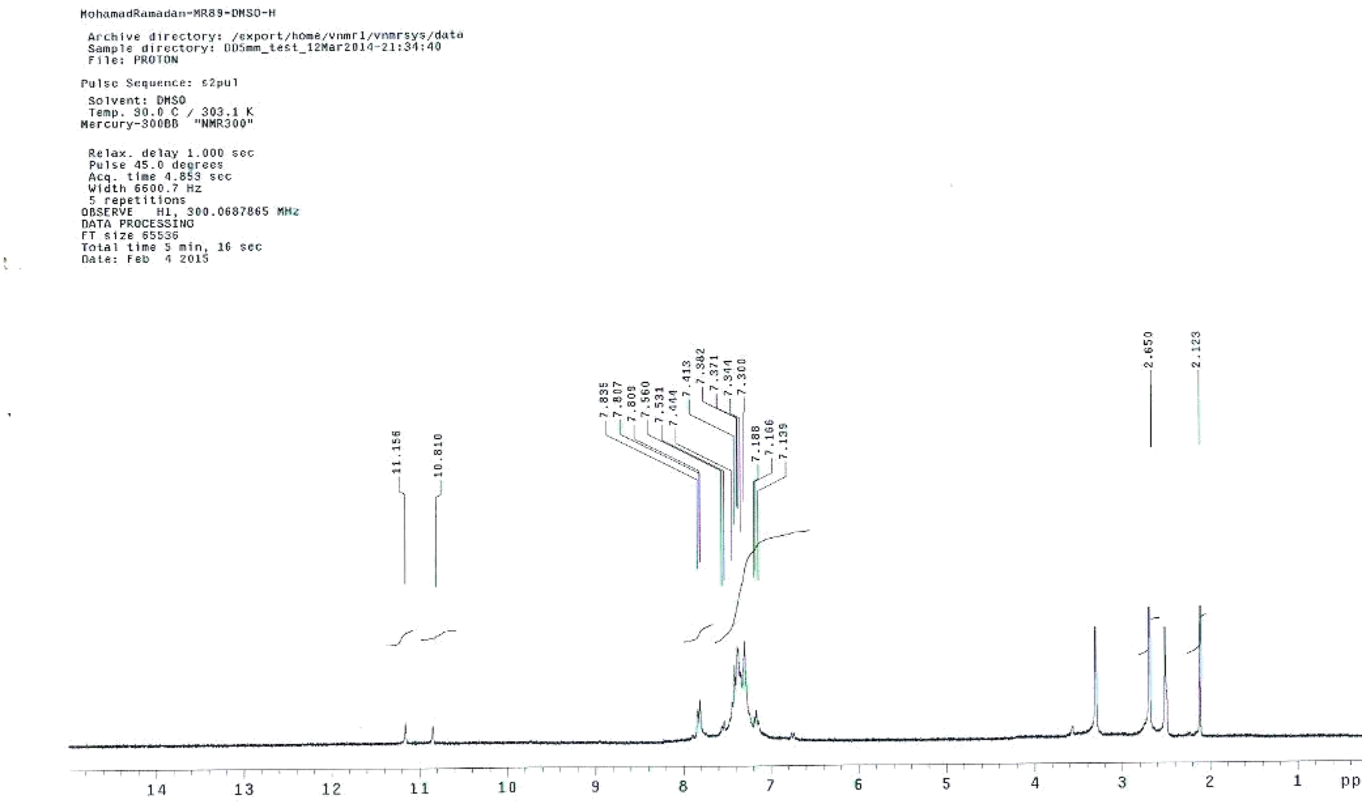

^{1}H NMR spectrum of compound 6h

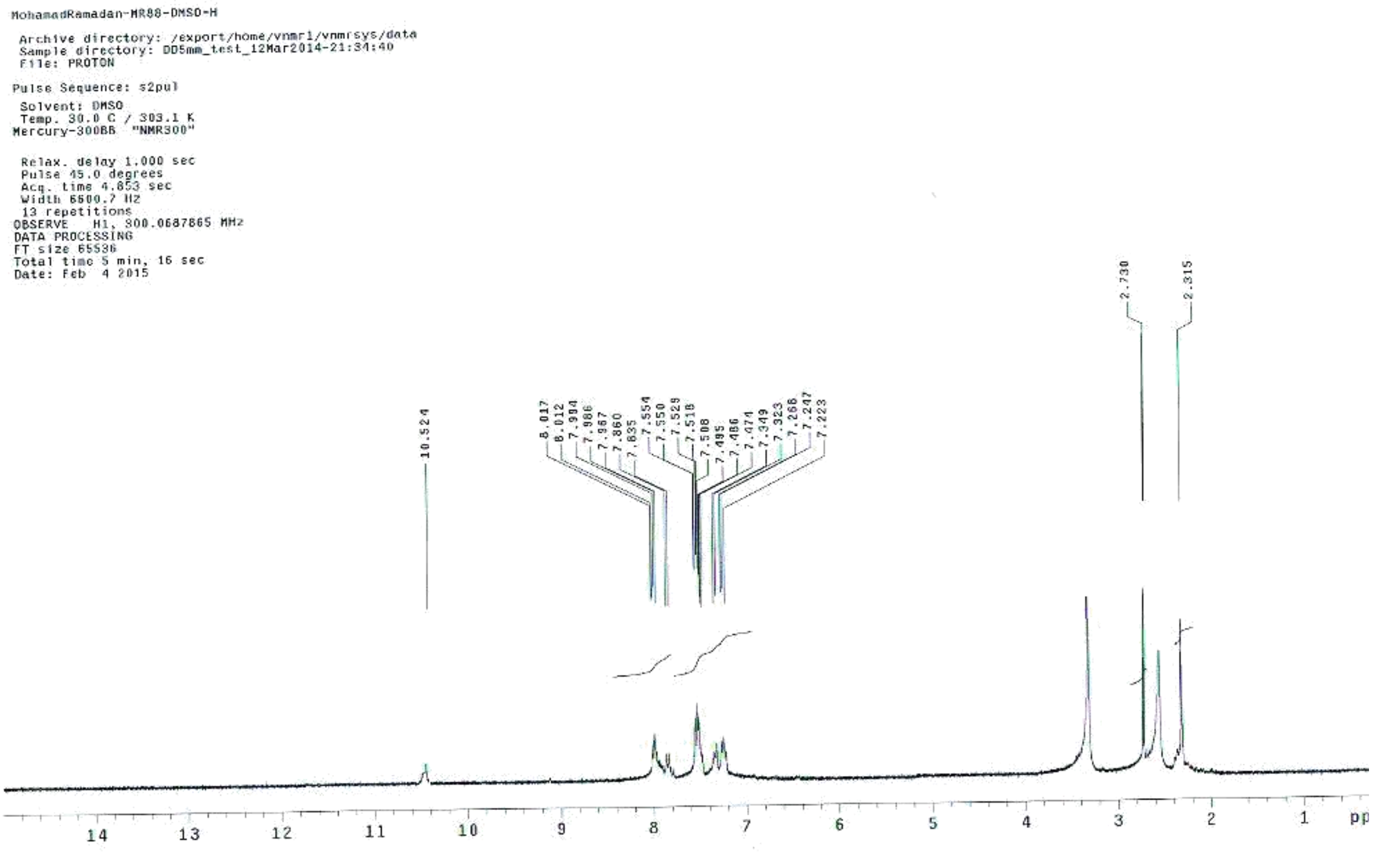

^{1}H NMR spectrum of compound 11a

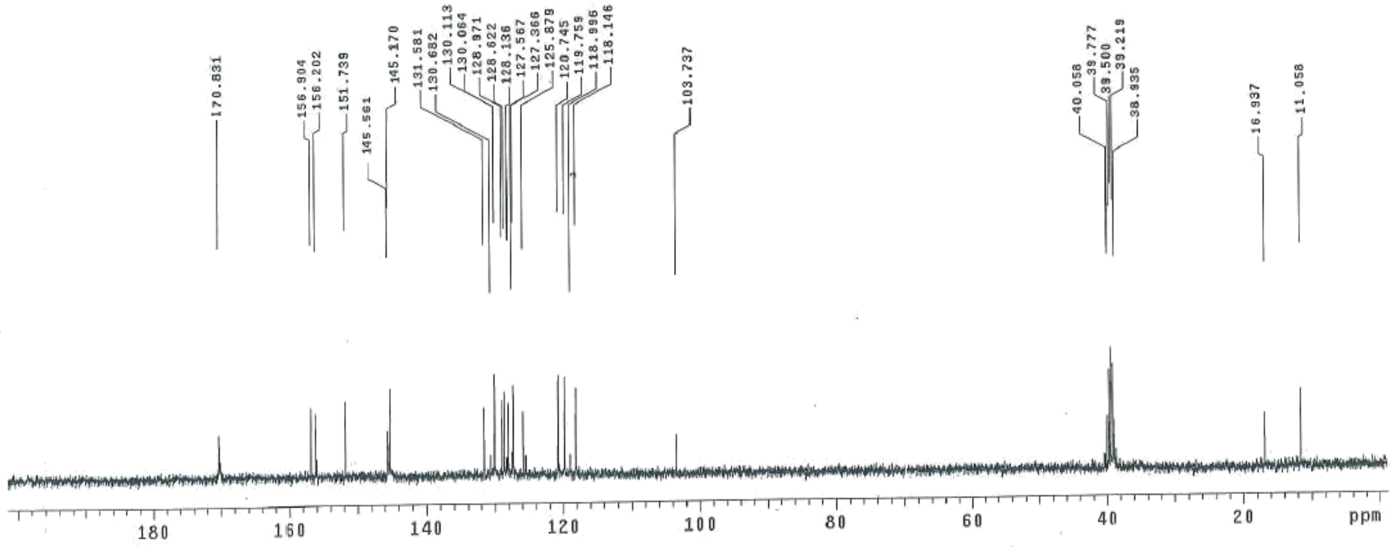

^{13}C NMR spectrum of compound 11a

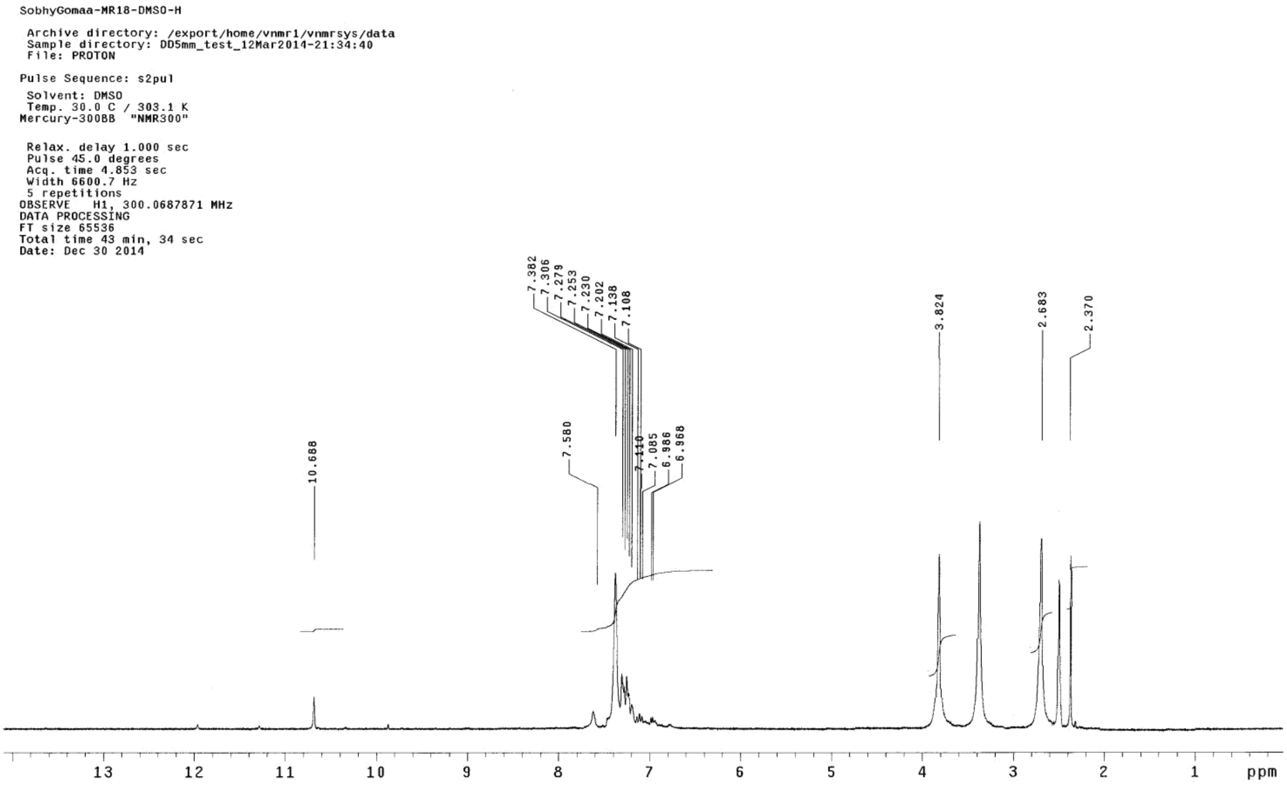

^{1}H NMR spectrum of compound 11e

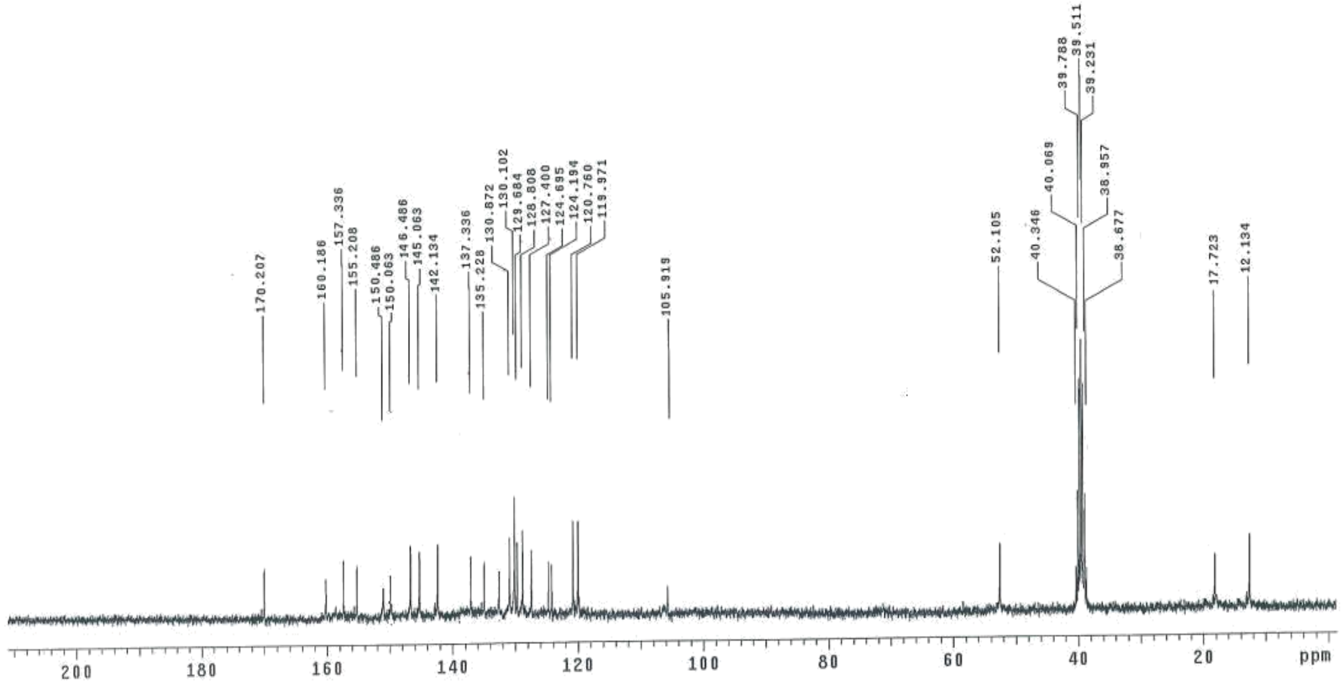

^{13}C NMR spectrum of compound 11e
